# mTOR Signaling Disruption and Its Association with the Development of Autism Spectrum Disorder

**DOI:** 10.3390/molecules28041889

**Published:** 2023-02-16

**Authors:** Shilu Deepa Thomas, Niraj Kumar Jha, Shreesh Ojha, Bassem Sadek

**Affiliations:** 1 Department of Pharmacology and Therapeutics, College of Medicine and Health Sciences, United Arab Emirates University, Al Ain P.O. Box 15551, United Arab Emirates; 2 Zayed Bin Sultan Center for Health Sciences, United Arab Emirates University, Al Ain P.O. Box 15551, United Arab Emirates; 3 Department of Biotechnology, School of Engineering and Technology (SET), Sharda University, Greater Noida 201310, India; 4 School of Bioengineering & Biosciences, Lovely Professional University, Phagwara 144411, India; 5 Department of Biotechnology, School of Applied & Life Sciences (SALS), Uttaranchal University, Dehradun 248007, India

**Keywords:** autophagy, autism spectrum disorder, PI3K/Akt/mTOR, tuberous sclerosis, PTEN, fragile X, phytoconstituents

## Abstract

Autism spectrum disorder (ASD) is a complex neurodevelopmental disorder characterized by impairments in social interaction and communication along with repetitive stereotypic behaviors. Currently, there are no specific biomarkers for diagnostic screening or treatments available for autistic patients. Numerous genetic disorders are associated with high prevalence of ASD, including tuberous sclerosis complex, phosphatase and tensin homolog, and fragile X syndrome. Preclinical investigations in animal models of these diseases have revealed irregularities in the PI3K/Akt/mTOR signaling pathway as well as ASD-related behavioral defects. Reversal of the downstream molecular irregularities, associated with mTOR hyperactivation, improved the behavioral deficits observed in the preclinical investigations. Plant bioactive molecules have shown beneficial pre-clinical evidence in ASD treatment by modulating the PI3K/Akt/mTOR pathway. In this review, we summarize the involvement of the PI3K/Akt/mTOR pathway as well as the genetic alterations of the pathway components and its critical impact on the development of the autism spectrum disorder. Mutations in negative regulators of mTORC1, such as TSC1, TSC2, and PTEN, result in ASD-like phenotypes through the disruption of the mTORC1-mediated signaling. We further discuss the various naturally occurring phytoconstituents that have been identified to be bioactive and modulate the pathway to prevent its disruption and contribute to beneficial therapeutic effects in ASD.

## 1. Introduction

Autism spectrum disorder (ASD) is defined as a diverse group of pervasive developmental disorders (PDDs) of neurodevelopmental origin characterized by the lack of social interaction skills, difficulty in communication, restricted and repetitive interests or behaviors, that show an early onset in childhood, starting at birth, and remain throughout adulthood resulting in lifetime persisting disabilities [1,2]. These social impairments in individuals with ASD may be related to their inability to interpret social signals, to guide appropriate behaviors. Potentially threatening situations, such as the proximity of others can trigger several physiological responses that help to maintain the distance between themselves and others during social interaction, showcasing the critical role of social signal interpretation in social interaction [3,4]. Investigations in healthy individuals indicate that neural activity in motor cortex is involved in behavioral regulation [5]. The stereotypical and repetitive behaviors are triggered as a result of neurochemical changes in the amygdala, cerebellum, hippocampus, and cerebral cortex [6]. In addition to the core features, other symptoms most commonly observed in kids include dyskinesia, sleep disorder, speech delay, gastrointestinal disturbances, anxiety, irritability, and epilepsy [7]. There are no treatments or cure available for autistic patients. The pharmacotherapy in ASD aims to decrease the non-core associated symptoms.

The neurodevelopmental theory suggests that the disruption of brain development in utero or early life may be associated with neuropsychiatric problems [8]. Environmental, genetic, epigenetic, and immunological factors contribute to the complex pathogenesis of autism spectrum disorder [8,9]. Various single-gene mutation disorders are linked to an augmented risk of developing autism spectrum disorder and include fragile X (FMR1), Rett Syndrome (MECP2), Timothy syndrome (CACNA1C), Angelman syndrome (UBE3A), and tuberous sclerosis (either TSC1 or TSC2) [10,11]. However, one common pathogenic molecular mechanism observed in the single-gene mutation disorders, such as fragile X and tuberous sclerosis, is the hyperactivation of the PI3K/Akt/mTOR intracellular signaling pathway and evidences suggest that the pathway plays a critical role in the development of ASD [12]. Preclinical studies in these disorders have revealed an increased activity of mTORC1 in the brain as well as ASD-related behavioral deficits. However, these effects were reversed by the mTORC1 inhibitor rapamycin [13]. Disruption in mTOR signaling cascade stimulates truncated translation in neuronal cells and leads to downregulation of protein synthesis at dendritic spines of the brain [13]. This irregular signaling leads to aberrations in brain structure, abnormal synapse formation, glial cell overexpression, neuroinflammation, oxidative stress, and mitochondrial dysfunction in the cerebellum, cerebral cortex, hippocampus and amygdala [14,15,16,17]. An increase in total brain volume and altered gray/white matter ratios are also seen in the autistic individuals [7]. Furthermore, alterations in GABAergic circuits and glutamate circuits have been seen in people with ASD [18].

Following the activation of the mTOR signaling pathway, there is an increase in the synthesis of proteins such as PSD-95, and presynaptic proteins such as synapsin 1 or the activity-regulated cytoskeleton-associated protein (Arc), which are crucial for synaptic plasticity processes such as the formation and maturation of new dendritic spines, memory processes, or long-term potentiation (LTP) [19]. Variations in the morphology of the dendritic spines, altered synaptic plasticity as well as impaired synaptic homeostasis are observed in the brain of ASD patients [20]. Autophagy is a vital process for synaptic formation and pruning during development [21]. Alterations in the mTOR pathway are related to numerous neurological disorders [22,23]. The PI3K/Akt/mTOR signaling cascade has been implicated in the pathogenesis of various neurodegenerative and neuropsychiatric disorders, e.g., Alzheimer’s disease [24], Parkinson’s disease [25], epilepsy [26], tuberous sclerosis complex (TSC) [11], fragile X syndrome [10], depression [27], schizophrenia [28] and bipolar disorder [29].

The various non-genetic elements mediating ASD risk comprise of parental age, maternal nutritional status, infections during pregnancy, and exposure to certain toxins, heavy metals, or drugs [30]. Clinical investigations reveal an imbalance in the microbiota of ASD individuals [31]. Gut microbiota dysbiosis has been implicated in ASD etiology. The gut–brain axis consists of a communication network that includes the vagal nerve, gut hormones, microbial metabolites, and the endocrine and immune systems, which control the gut process and link it to the brain [32]. Microglial activation and neuroinflammation caused by the gut microbiota are implicated in the pathological process in individuals with ASD. Modulation of the gut microbiota has been found to improve ASD-related behavioral symptoms [33]. Infections in the course of pregnancy activate the maternal immune system that can lead to social behavior changes in the offspring [32].

Evolving evidence suggests that disturbances in the circadian clock trigger irregularities in the mTOR signaling pathway [20]. Investigations have revealed that that disruption of circadian rhythms during neurodevelopment can lead to hyperactivation of the mTOR and MAPK signaling pathways along with behavioral deficits, and impairment in social interaction and communication [34]. Traditional medicine has been commonly used in the treatment of ASD. Plant bioactive constituents with neuroprotective actions have therapeutic potential in the treatment of neuropsychological disorders [35,36]. This review discusses the amassing literatures on ASD, predominantly focusing on the dysregulation of PI3K/Akt/mTOR signaling cascade as well as the genetic alteration of the pathway components in the development of ASD. In addition, it also summarizes studies examining and showcasing the use of various phytoconstituents found to be effective in preventing the progression of ASD.

## 2. Aims and Methods

This review helps to summarize the main research outcomes regarding the implications of the PI3K/Akt/mTOR pathway as well the various genetic alterations of the pathway components in the etiology of autism. Over one hundred references were retrieved from PubMed, Scopus, and Google Scholar using relevant keywords in combination. Articles published over the years were reviewed by performing a search using the following keywords (“autism” OR “autism spectrum disorder”) and (“PI3K” OR “Akt” OR “mTOR” OR “PTEN” OR “TSC”) in [title/abstract/keywords]. Papers related to the topic were identified. Data were obtained from inception until December 2022, with a focus on English language papers. By excluding nonrelevant full texts, 165 reports were included in the systematic part of this review.

## 3. P13K/Akt/mTOR Pathway

### 3.1. The Structure and Components of mTOR

The kinase TOR was originally recognized in the yeast Saccharomyces cerevisiae with TOR1 and TOR2 genes encoding for Tor 1 and Tor 2 isoforms [37]. Rapamycin is a macrolide antibiotic obtained from the soil bacterium Streptomyces hygroscopicus that inhibits mTOR [38]. Initially developed as an antifungal drug, rapamycin also possesses immunosuppressive and anti-proliferative properties [39]. Rapamycin complexes with FK506-binding protein 12 (FKBP-12), to inhibit the target of rapamycin (TOR) and exerts its actions. In mammals, TOR kinase, also identified as mammalian TOR or mTOR, is ubiquitously expressed in all cell types. mTOR, belonging to the family of phosphatidylinositol kinase-related kinase (PIKK) [40] is a well-conserved PI3-kinase and occurs as two distinct protein complexes, mTOR Complex 1 (mTORC1) and mTOR Complex 2 (mTORC2) [41,42]. The sensitivity towards rapamycin is more for mTORC1 than mTORC2 [43]. mTORC1 comprises of the components Raptor (regulator-associated protein of the mTOR, necessary for the activation of the complex), Deptor (DEP domain-containing mTOR interacting protein), mLST8 (mammalian lethal with Sec13 protein 8, also termed as GβL), PRAS40 (proline-rich Akt substrate of 40 kDa, a negative regulator of mTORC1, and Ttil (telomere maintenance 2 (Tel 2) interacting protein 1 complex that stabilizes mTOR and produces a scaffold for recruitment of substrates) [44,45]. mTORC2 consists of Rictor (rapamycin insensitive companion of mTOR), Deptor, mLST8, mSIN1 (the mammalian stress-activated protein kinase interacting protein), and Protor (the regulatory subunit protein observed with rictor 1 and 2) [46]. Deptor negatively regulates mTORC1 and mTORC2 by inhibiting substrate binding. Both the complexes have specific substrate preferences and produce different downstream signaling actions to modulate cellular function [47]. Upon activation, mTORC1 exerts downstream biological effects consisting of mRNA translation via the phosphorylation of 4E-BP1 and p70S6 Kinase, autophagy inhibition, and ribosome biogenesis. mTORC2 phosphorylates SGK1 to regulate growth and activates protein kinase C to control cytoskeletal dynamics [48].

At the amino terminal part, mTOR comprises of 20 tandem HEAT (Huntington, Elongation Factor 3, Protein Phosphatase 2A, TOR1) repeats that assist in protein–protein interactions followed by a FAT (FRAP, ATM, TRAP) domain and kinase domain (KD) with two lobes: the N- and C-lobes [43]. The FRB domain (FKBP/rapamycin-binding domain), is in the KD N-lobe, whereas KD C-lobe contains LBE and acts as a binding site for mLST8. The extreme carboxyl terminal of mTOR contains FATC, another FAT domain [49]. The FAT and FATC domain regulate actions of the kinase. Raptor interacts with the HEAT repeats of mTOR in addition to binding with its extreme carboxyl terminal, whereas PRAS40 binds to Raptor. The binding of Rheb-GTP to the HEAT domain leads to the activation of mTORC1 kinase [24].

mTORC1 responds to nutrient signals such as glucose and amino acids, growth factors and metabolic factors, and controls biological functions including lysosome biogenesis, autophagy, protein synthesis, and energy metabolism [50]. When cellular energy levels are low, AMPK by the direct phosphorylation of Raptor impedes mTORC1, disrupting the mTORC1 complex [51]. mTORC2 is unresponsive to nutrients but responds to growth factors and plays a significant role in cell growth, cell proliferation, and motility [26,52]. High concentrations of cellular nutrients activate mTORC1. In contrast to mTORC1, mTORC2 activity is augmented during activation of the TSC1/TSC2 complex (tuberous sclerosis complex 1/2). Growth factors can activate mTORC2 through the PI3K signaling pathway. mTORC2 contributes to cellular proliferation and cellular metabolism by regulating actin through PKC-α and Rho GTPase [53]. mTORC1 is positively regulated by Akt, the Akt phosphorylation by mTORC2 stimulates the actions of mTORC1, thereby inhibiting autophagy [54].

TSC acts as a GTPase-activating protein (GAP) thereby converting G protein Rheb (Rheb-GTP) into inactive GDP-bound form (Rheb-GDP). Active Rheb-GTP, interacts with Raptor and controls the binding of 4EBP1 to mTORC1, thus increasing the activity of mTORC1. During hypoxia, AMPK leads to transcriptional regulation of DNA damage response 1 (REDD1/RTP801) expression to regulate the activity of TSC1/TSC2 and inhibit mTORC1 by promoting the release of TSC2 from its inhibitory binding to protein [43].

### 3.2. Activation of mTOR Signaling

The PI3K/Akt/mTOR signaling pathway is essential for numerous cellular functions including autophagy. The cell growth and survival are primarily regulated by mTOR in response to nutrients and other growth stimuli [55]. A significant role of mTOR activity is also observed in synaptic plasticity, axonal guidance, neuronal recovery, and consolidation of memory [56]. mTOR is recognized as the master regulator of protein synthesis through its interaction with several upstream signaling molecules.

The phosphoinositide 3-kinases (PI3-K) and protein kinase B (Akt/PKB) are the upstream signaling molecules of mTOR, constituting the PI3K/Akt/mTOR pathway, and are involved in regulation of autophagy [47]. The binding of growth factors and insulin to tyrosine kinase receptors activates the kinase PI3K. This results in the phosphorylation of phosphatidylinositol-4,5-phosphate (PIP2) to generate phosphatidylinositol-3,4,5-triphosphate (PIP3) [57]. The second messenger PIP3 recruits Akt and promotes its phosphorylation by PDK1 and mTORC2 on the Thr308 and Ser473 sites, respectively. This emerges through the phosphorylation of TSC, impairing its inhibitory activity on mTOR [58]. TSC, a heterodimer, consists of hamartin (TSC1) and tuberin (TSC2). Hence, the activation of PI3K/Akt signaling via inhibition of TSC promotes mTORC1activity [25]. PTEN (phosphatase and tensin homolog) is a PI3K antagonist. PTEN, a phosphatase acts antagonistically to the kinase (PI3K) in conversion of PIP2 to PIP3, thus acting as a crucial negative control of incoming signals [59]. Table 1 depicts the various genes of the PI3K-Akt-mTOR pathway implicated in ASD.

**Figure 1:**
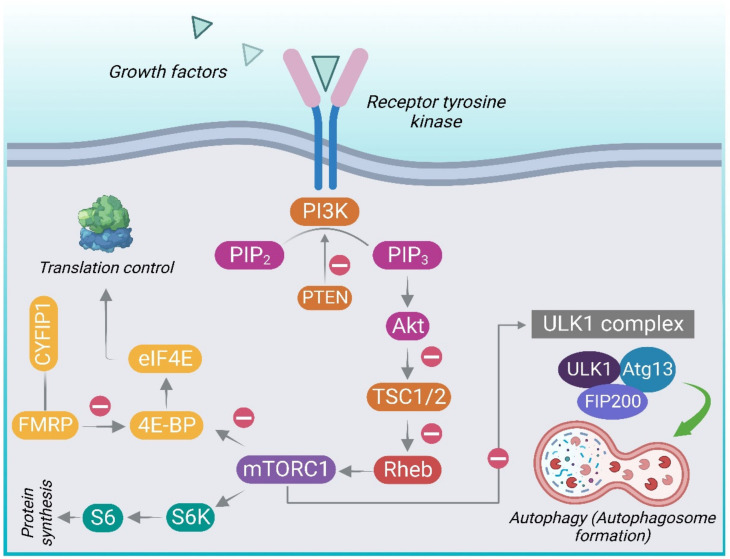
An overview of the PI3K/Akt/mTOR intracellular signaling pathway. Different extracellular stimuli, e.g., growth factors and insulin, mediate their effects by binding to receptors belonging to the receptor tyrosine kinase (RTK) family. This leads to activation of phosphatidylinositol-4,5-bisphosphate 3-kinase (PI3K). PI3K converts phosphatidylinositol (3,4)-bisphosphate (PIP2) into phosphatidylinositol (3,4,5)-trisphosphate (PIP3), which recruits Akt-kinase and stimulates the Akt phosphorylation by PDK1 and mTORC2, on the Thr308 and Ser473 sites, respectively. The activation of the PI3K/Akt pathway through the inhibition of TSC (tuberous sclerosis complex) leads to the activation of mTORC1. Inhibition of the tuberous sclerosis complex (TSC) results in the loss of its ability to convert the Ras homolog enriched in brain active form (RhebGTP) to the inactive GDP-bound form (Rheb-GDP) leading to the subsequent activation of mTORC1. PTEN acts antagonistically to PI3K in conversion of PIP2 to PIP3, functioning as an important negative control. Activation of mTORC1 leads to phosphorylation of p70 ribosomal protein S6 kinase (p70S6K) and 4EBP1 (eukaryotic initiation factor 4E-binding protein) which, in turn, phosphorylates the ribosomal protein S6 leading to protein translation and synthesis. Active mTORC1 blocks autophagy by inhibitory phosphorylation of the ULK1 complex comprising of autophagy-related proteins (UNC51-like kinase 1 (ULK1), the autophagy-related gene 13 (Atg13), and FAK-family interacting protein of 200 kDa (FIP200). 4E-BP1 prevents translation initiation by sequestering eukaryotic translation initiation factor 4E (eIF4E). mTOR phosphorylates the eukaryotic initiation factor 4E-binding protein 1 (4E-BP1), thereby preventing it from binding to eIF4E, thus liberating eIF4E to enable translation. FMRP (fragile X mental retardation protein) is the protein produced by FMR1gene. FMRP binds to CYFIP1 (cytoplasmic FMRP-interacting protein 1) to form FMRP-CYFIP1 complex that obstructs translation by binding to eIF4E, which is obliterated in fragile X syndrome.

Activation of mTOR leads to phosphorylation of S6K1 (p70 ribosomal protein S6 kinase, p70S6K) and 4E-BP1 (eukaryotic initiation factor 4E-binding protein) which, in turn, phosphorylates the ribosomal protein S6 leading to protein translation and synthesis [50,60]. 4E-BP1 prevents translation initiation by sequestering eukaryotic translation initiation factor 4E (eIF4E). mTOR phosphorylates 4E-BP1, lowering its affinity for the eukaryotic initiation factor 4E (eIF4E), thus liberating eIF4E to facilitate translation. Hence, mTOR primarily controls protein translation through its downstream targets [61]. Figure 1 gives an overview of the PI3K/Akt/ mTOR signaling pathway.

To regulate the activity of mTORC1 and reinstate TSC regulation, the mTORC1 substrate S6K1, as a part of a negative feedback loop, directly phosphorylates insulin receptor substrate 1 (IRS-1), preventing further insulin-mediated PI3K/Akt/mTOR pathway activation [59]. Concurrently, active mTORC1 blocks autophagy by inhibitory phosphorylation of the ULK1 complex comprising of autophagy-related proteins (UNC51-like kinase 1 (ULK1), the autophagy-related gene 13 (Atg13), and FAK-family interacting protein of 200 kDa (FIP200) [62]. By phosphorylating the ULK1 complex, mTORC1 inhibits initiation of autophagosomes [63]. However, upon deprivation of nutrients, adenosine monophosphate-activated protein kinase (AMPK) phosphorylates mTORC1 and activates ULK1 followed by initiation of autophagy, activated ULK1 phosphorylates, and activates Beclin-1, triggering the activation of the VPS34 (vacuolar protein sorting 34) complex [64]. The latter leads to accumulation of phosphatidylinositol-3-phosphate in the phagophore, resulting in the sequential recruitment of numerous binding proteins including ATG5-ATG12-ATG16L1 [60]. This trimeric complex along with light chain 3 (LC3) is crucial for membrane elongation. After the formation of autophagosome, it fuses with the lysosome to form an autolysosome and continues with macromolecule degradation [52]. The gene AMBRA1 (Activating Molecule in Beclin 1-Regulated Autophagy) displays sex-differential expression and has been related to autism and schizophrenia-related phenotypes, among females, in both humans and mice [65]. AMBRA1 is a positive regulator of Beclin1 and is a prime component in autophagosome formation. It is involved in neurodevelopment and autophagy. Heterozygous deficiency of AMBRA1 can lead to autistic behavior in a sexually dimorphic manner.

## 4. Role of PI3K/Akt/mTOR in Autism Spectrum Disorder

**Figure 2:**
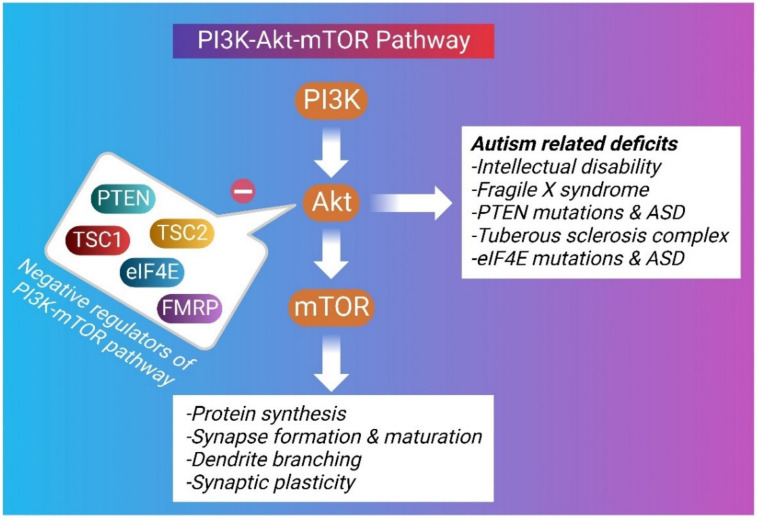
A graphical depiction of the inter-relationship of the PI3K-mTOR system and autism-related deficits.

Autism is known as an early-onset disorder of the developing CNS. Diverse anatomical abnormalities in brain structure and cellular changes have been observed in autistic brains, signifying a complicated and multifactorial etiology behind ASD [66]. Microcephaly, macrocephaly, enlarged brain size, increased cell density, decreased number of Purkinje cells in the cerebellum, cortical dysgenesis, and migration abnormalities have been observed in the brains of autistic individuals [66]. A disruption in synaptic pruning has also been detected in ASD [67]. The increased spine density in ASD brains has been correlated with increased phosphorylation of the mTOR as well as its downstream effectors, ribosomal protein S6 [68]. The Akt/mTOR pathway is crucial for the process of learning and memory formation by enhancing LTP of the synapses [69]. Inhibition of the mTOR activity has been found to increase the PI3K/Akt/mTOR-mediated autophagic pathway and improve social interactions in valproic acid-induced ASD [70,71]. Several ASD candidate genes have been identified through whole-genome linkage studies, copy number variation screening, SNP analyses, and genome-wide association studies. These potential gene targets, include the Akt/mTOR signaling cascade and its downstream effectors comprising of FMR1, PTEN, TSC1, and TSC2 [72]. The Akt/mTOR pathway regulates several cellular processes that influence neurodevelopment and may be relevant to the development of ASD symptoms [12,73] (Figure 2).

Wang et al. investigated the effect of fetal exposure to valproic acid in male offspring mice. The outcomes of the study revealed hyperactivation of the PI3K/Akt/mTOR signaling pathway and reduced expressions of the synaptic proteins PSD95 and p-Syn, dendritic spine damage, and improper synaptic development in the prefrontal cortex, ultimately leading to the development of ASD-like phenotype [74]. Inhibition of mTOR activity by mTOR antagonist rapamycin was found to increase the PI3K/Akt/mTOR-mediated autophagic pathway and improve social interaction in valproic acid-induced ASD [70]. In another study, valproic acid-exposed mice exhibited deficits in early postnatal development, however, these behavioral deficits and autophagic dysregulation were abolished by postnatal administration of rapamycin [71].

### 4.1. Fragile X Syndrome

Absence of the fragile X messenger ribonucleoprotein 1 (FMRP) causes ASD and intellectual disability, commonly referred to as the fragile X syndrome [10]. FXS is characterized by ASD, intellectual disability, anxiety, and physical features, such as macrocephaly and macroorchidism in males [13]. The genetic defect observed in FXS subjects is due to the elongation of CGG repeats in the 5′-untranslated region of the FMR1 gene, leading to reduced expression of the gene. FMRP (fragile X mental retardation protein), the protein produced by the FMR1 gene, suppresses the translation of postsynaptic components of neurons. FMRP binds to CYFIP1 to form FMRP-CYFIP1 complex that binds to eIF4E and prevents translation initiation, which is obliterated in FXS. Uninhibited eIF4E-mediated initiation of translation may lead to the progression of ASD in FXS [75]. FMRP is essential in synaptic function and neuronal plasticity through its interaction with pre- and postsynaptic proteins important for neurotransmission and structure [76]. It also binds to the AMPA glutamate receptor subunits (GluR1 and GluR2) [77].

Fmr1 KO mice display significant behavioral abnormalities along with cognitive deficits in different behavioral tasks [78]. Fmr1 KO adult mice exhibit increased spine density and length, as well as increased immature, thin spines [79]. Fragile X mental retardation protein (FMRP), encoded by the FMR1 gene, negatively regulates the synthesis of post-synaptic glutamate receptors [80]. Additionally, LTD triggered by mGluR was enhanced in Fmr1 KO mice [81]. The role of metabotropic glutamate receptors (mGluRs) in the pathophysiology of FXS were investigated by Thomas et al. Phenotypic alterations such as memory impairment and extreme hippocampal protein synthesis seen in Fmr1 knockout mice were shown to be prevented by the reduced expression of mGlu5. The inhibition of mGlu1 and mGlu5 receptors efficiently decreased repetitive behavior in Fmr1 knockout mice with better results observed with mGlu5 inhibition in comparison to mGlu1 inhibition in enhancing the motor learning [82]. Furthermore, investigations conducted by Sare et al. demonstrated that the long-term administration of rapamycin did not bring about any improvements in the behavioral deficits in the Fmr1 knockout mice [83]. These investigations further suggest that mGlu5 receptors contribute significantly to the pathogenesis of the disease, which can have noteworthy therapeutic consequences for fragile X and related developmental disorders.

Multiple investigations recommend a link between the deletion of FMRP and dysregulation of mTROC1 in FXS. An increase in the levels of phosphorylated mTOR, S6K1, Akt as well as initiation factor 4E (eIF4E) has been observed in the protein lysates of individuals with fragile X syndrome [84]. Deficiency of FMRP leads to enhanced activity of PI3K and Akt in Fmr1 KO mice, implicating the pathological role of a hyperactivated PI3K/Akt signaling pathway [85]. The hippocampus in Fmr1 knockout mice has been shown to possess an increased rate of protein synthesis in addition to the hyperactivation of the mTORC1 pathway [86,87]. Augmented expression of the FMRP binding protein CYFIP1 is observed in Fmr1 KO mice, further linking FXS to excess protein synthesis via eIF4E [88]. Furthermore, investigations by Bhattacharya et al. showed that genetic reduction of S6K1 prevented exaggerated protein synthesis and enhanced mGluR-dependent long-term depression (LTD) in FXS model mice. S6K1 deletion prevented immature dendritic spine morphology and multiple behavioral phenotypes, including social interaction deficits and ASD-related behavioral deficits [89].

Synaptic homoeostasis is essential for the normal function of the brain. Multiple evidence has revealed increased dendritic spine density and aberrant dendritic spine morphology in subjects with ASD. Investigations by Yan et al. established decreased autophagy and down-regulation of LC3-II in hippocampal neurons of rodent models of human FXS [90]. The outcomes of the study further demonstrated that mTOR hyperactivation led to reduced autophagy inducing spine defects, aberrant synaptic plasticity and impaired cognition in Fmr1-KO mice. However, activation of autophagy corrected the abundance of proteins implicated in spine structure (postsynaptic density protein, PSD-95) and synaptic plasticity (activity-regulated cytoskeletal-associated protein, Arc/Arg3.1) in neurons lacking fragile X mental retardation protein as well as anomalous dendritic spine morphology and metabotropic glutamate receptor-dependent long-term depression (mGluR-LTD). Furthermore, the involvement of autophagy was exhibited as the Fmr1^−/−^ neurons displayed an accretion of ubiquitinated protein aggregates [90]. These outcomes suggest that hyperactivation of mTORC1 result in deficits in autophagy and social behaviors.

### 4.2. PTEN in ASD

A subset of individuals with ASD and extreme macrocephaly exhibit mutations in the PTEN gene, referred to as “macrocephaly/autism syndrome.” They are recognized as a spectrum called PTEN hamartoma tumor syndrome that result from PTEN gene mutations [13]. Nearly 7–17% of ASD individuals with macrocephaly and 1–5% of those with ASD have a mutated PTEN gene. The suppression of hyperactive Akt/mTOR in mice deficient for PTEN and TSC1/2 have resulted in improvement of ASD-associated symptoms. PTEN mutations are involved in several brain disorders, including mental retardation, seizures, and ASD. PTEN, phosphatase, and tensin homolog act antagonistically to PI3K in conversion of PIP2 to PIP3 [59]. PTEN is a main inhibitor of the PI3K/Akt pathway with downstream effects on mTOR signaling. PTEN deletion in animal models leads to hyperactivation of the PI3K/Akt/mTOR signaling pathway in the hippocampal regions resulting in long-term alterations in social behaviors, repetitive behavior, and anxiety similar to autistic phenotypes establishing a role of mTOR pathway in ASD [91]. Inhibition of PTEN function can have profound and manifold effects on neuronal cells contributing to the development of autistic behaviors [92]. PTEN gene deletion in the cerebral cortex and hippocampus of mice leads to progressive macrocephaly, increased spine density, and synaptic abnormalities with increased presynaptic varicosities. These effects can be attributed to the overactivation of the PI3K/Akt/mTOR signaling pathway leading to an increase in Akt and S6 phosphorylation in the brain [93]. Increased soma size and axonal growth as well as hypertrophic and ectopic axonal projections were some neuronal alterations seen in PTEN KO mice.

Investigations have revealed a dramatic increase in dendrite size and enlarged hippocampus with absence of PTEN. Loss of PTEN also leads to anomalous LTP and LTD [91]. Lugo et al. investigated the behavioral and molecular significance of PTEN deletion in mice. The PTEN knockout mice showed deficits in social chamber and social partition test. Repetitive behavior and anxiety were also observed. PTEN deletion significantly downregulated the mGluR signaling in the hippocampus whereas an increase in total and phosphorylated fragile X mental retardation protein was observed [91]. Mutations in the genes in the PI3K-Akt-mTOR signaling pathway, comprising PIK3CA, PTEN, mTOR, and PPP2R5D have been reported in patients with autism spectrum disorder/development delay and macrocephaly [94].

PTEN knockout mice develop behavioral abnormalities suggestive of human autism, with diminished learning, social interaction deficits, seizures, and anxiety-like behaviors [95,96]. These preclinical investigations further establish the importance of PTEN in the pathogenesis of macrocephaly and ASD-related cognitive deficits and abnormal behavior. Further, Tai et al. demonstrated that the tau protein aids autism-like behaviors, and even partial reduction of tau prevented ASD-like behaviors and related neural abnormalities in Cntnap2^−/−^ mice models [97]. Tau protein suppresses PTEN activity via interaction mediated by its proline-rich domain. The reduction in tau protein was found to prevent PI3K overactivation and megalocephaly. The recovery of the behavioral deficits observed in the PTEN mutant mice is associated with mTOR inhibition. Zhou et al. reported that the mTORC1 inhibitor, rapamycin, prevented these behavioral abnormalities, providing evidence that the mTORC1 pathway downstream of PTEN is crucial for this phenotype [98]. Chronic administration of rapamycin prevented brain enlargement and neuronal soma hypertrophy, and alleviated axonal and dendritic hypertrophy.

PTEN genetic mutations are highly predominant in developmental delays and mental problems [99]. PTEN germline mutations have been detected in a subsection of children with ASD/macrocephaly [100]. In patients with PTEN-ASD, prominent white matter and cognitive abnormalities with reduced processing speed and working memory deficits have been observed [101]. Furthermore, neurobehavioral evaluations conducted in patients with PTEN-linked ASD suggest primary disruption of frontal lobe systems with severe cognitive dysfunction, slow reaction time, attention deficits, and decreased memory processing capability [96]. Rapamycin treatment in PTEN knocked out oligodendrocyte and Schwann cells was found to reduce the hypertrophy of white matter [102]. Mutations in certain postsynaptic cell adhesion molecules like neuroligins have been found to affect protein synthesis. Neuroligins are vital in synaptic transmission and synaptogenesis. In Nlgn3KO mice, upregulation of Akt/mTOR signaling resulted in augmented protein synthesis and dendritic growth [103]. Further treatment of neurons with either mTOR inhibitor, rapamycin or PI3K/Akt activity inhibitor, LY294002 prevented the morphological abnormalities in knockout models. The study also established a new relationship between Akt/mTOR signaling cascade and NL3. The hyperactivation of Akt/mTOR signaling with NL3 defects was mediated by decrease in PTEN expression and interaction of MAGI-2, a scaffold protein, with both PTEN and NL3. These preclinical and clinical investigations delineated above strongly suggest that hyperactive mTOR due to mutations in PTEN result in the development of ASD and other cognitive deficits.

### 4.3. TSC in ASD

TSC1 and TSC2 form a heterodimeric complex (TSC1/2) that receives signaling inputs from protein kinase B, Erk1/2, AMPK, and GSK3β functioning as a signaling node that can modulate the activity of the mTORC1 [104]. Tuberous sclerosis complex (TSC) is a genetic disorder that develops from mutations of the TSC1 and TSC2 gene. The haploinsufficiency of TSC1 or TSC2 weakens the inhibition of mTORC1. This is followed by mTORC1-dependent increased phosphorylation of S6, S6Ks, and 4E-BPs resulting in altered protein synthesis. All organ systems are affected by TSC, but involvement of the central nervous system presents early [105]. A rising concern among clinicians is the high prevalence of TANDs (TSC-associated neuropsychiatric disorders) in the TSC population. TAND spectrum is wide-ranging and consists of cognitive, behavioral, and psychiatric conditions such as attention deficit hyperactivity disorder (ADHD), autism spectrum disorder (ASD), depression, and anxiety [106]. TSC is one of the most common causes of syndromic ASD [107].

Deficits in cognition, memory, and social behavior have been observed in haploinsufficient models of Tsc1^+/−^ and Tsc2^+/−^ mice in the absence of neuropathology and seizures [108]. The omission of TSC2 in cerebellar Purkinje cells resulted in the development of ASD-related social deficits in mice, further signifying that the cerebellar-specific mTOR signaling regulates mouse social behavior [109]. Furthermore, the social interaction deficits and behavioral abnormalities observed in rodent models of TSC were ameliorated upon treatment with rapamycin [109,110]. These preclinical investigations establish the therapeutic utility of mTOR blockade in neurological manifestations in TSC.

Post-mortem investigations in temporal lobe tissue from autistic individuals demonstrated augmented dendritic spine density in addition to decreased synaptic pruning. This could be attributed to overstimulation of mTOR and impaired autophagy, as evidenced by lower levels of LC3-II and higher p62. This presents an association between mTOR hyperactivation, consequent autophagic dysfunction, and decreased synaptic pruning in autistic individuals [111]. Furthermore, investigations in Tsc2^+/−^ mice, rapamycin treatment was found to improve ASD-like behaviors and ameliorated spine-pruning defects, however, the effects were absent in double mutant Tsc2^+/−^: Atg7cKO mice, highlighting the importance of autophagic pathways to reverse the pathology [111]. The TSC1/2 complex is essential for the regulation of synaptic function and neuronal morphology in the brain. Hyperactivation of mTORC1 and enhanced downstream activity due to TSC1/TSC2 mutations can lead to neuronal dysfunctions and impaired axon regulation, abnormal dendritic morphogenesis, and synapse formation [112]. Post-mortem investigations in individuals with autism spectrum disorder (ASD) have revealed an augmented density of excitatory synapses in their brains which could be attributed to aberrant mTOR-dependent synaptic pruning. Further, in silico modeling in TSC2 haploinsufficient mice has demonstrated mTOR-dependent enhanced spine density linked with ASD-like stereotypes and cortico-striatal hyperconnectivity [113]. However, the administration of mTORC1 inhibitor, rapamycin, reversed these effects (Pagani et al., 2021). This strongly suggests a positive correlation between connectivity and spine density, which is, in turn, channeled by mTORC1. These studies further confirm that the omission of TSC1 or TSC2 or variations in the TSC-related cell signaling significantly increase an individual’s risk of ASD development [114]. The reversal of learning deficits, autistic-like deficient social approach, and molecular abnormalities by rapamycin, further demonstrates the therapeutic implication of mTOR signaling cascade in TSC-associated neurological problems [109,110,115].

### 4.4. The Excitatory/Inhibitory Imbalance

Autophagy establishes an association between mTOR hyperactivity, neuronal excitation/inhibition equilibrium and ASD-like behavior [116]. One of the key mechanisms underlying the pathogenesis of the neurodevelopmental disorders, such as ASD, is the loss of balance between the excitatory (E) and inhibitory (I) activity in the brain circuits [18,117]. The excitatory/inhibitory balance in the brain is regulated by glutaminergic pyramidal neurons and GABAergic interneurons [118]. Low levels of GABA, or reduction in GABAergic interneurons, as well as inhibition of GABAergic innervations has been observed in the patients with ASD as well as rodent models thereby altering the E/I balance. Neurexins and neuroligins are presynaptic and postsynaptic binding proteins that coordinate to form transsynaptic complexes. These proteins directly facilitate synapse formation and stabilization [119]. Neuroligins like NLGN-1, NLGN-3, and NLGN-4 localize to the glutamate postsynaptic membrane, whereas NLGN-2 localizes primarily to GABA synapse. Neuroligins participate in the formation of glutamatergic and GABAergic synapses. Mutations in neuroligins and neurexins bring about changes in synaptic structure and plasticity. Genomic studies have identified alterations in neurexins and neuroligins in ASD [120].

The levels of glutamate and GABA vary in autistic children. The abrupt synaptic connectivity is related to the alterations in expression and function of glutamate receptors subsequently modulating neuronal function [80]. Altered functioning of the metabotropic glutamate receptor mGluR5 is observed in fragile X syndrome, intellectual disability, and autism [121]. Group I mGluRs are associated with NMDA receptors and regulate the NMDA receptor-mediated LTP and LTD. Tripartite motif (TRIM) 32, a maintainer of mTOR, plays an essential part in the ubiquitin-protease degradation of proteins and is mainly expressed on neural progenitor cells (NPCs) in the nervous system [122]. Rare copy number variation analysis has demonstrated a strong association between the absence of TRIM32 gene and autistic behavior signifying the essential role played by TRIM32 in the brain development [123]. Consistent with this outcome, knockout of TRIM32 gene leads to hyperexcitability and ASD-like behavior in addition to impaired generation of GABAergic neurons and dysfunctional GABAergic inhibition [124]. Zhu et al. demonstrated that TRIM32 regulates the generation of GABAergic interneurons. TRIM32 sustains activity of mTOR through promoting the degradation of a GTPase activating protein, RGS10 (regulator of G protein signaling protein 10). mTOR activity is suppressed by RGS10 via accelerating the hydrolysis of GTP from Rheb which is inhibited by the transplantation of L/MGE progenitors or treatment with a GABAA receptor agonist [124].

Preclinical studies have revealed that autophagy affects social behavior after postnatal development. Autophagic deficiency induced by Atg7 deletion in GABAergic interneurons and excitatory neurons of the medial PFC in adolescent mice caused autistic-like behavioral abnormalities including deficits in social interaction and increased anxiety [125]. The suppression of autophagy either due to hyperactivation of mTOR or mutation in autophagy genes, interrupts GABAA receptor expression leading to a reduction in inhibitory inputs, followed by neuronal hyperexcitability. Different therapeutic approaches have been used to restore E/I imbalance, such as treatment with mGluR5 antagonist, NMDAR agonist, and GABAR agonist. Investigation in rodent models from FXS have revealed variations in GABA-mediated synaptic transmission, further suggesting therapeutic potential of GABA receptor agonism in FXS [119].

### 4.5. Inflammatory Mechanisms in ASD

The levels of inflammatory molecules such as cytokines, IL-1β, IL-6, and IL-8 have been found to increase in the brain, CSF, and peripheral blood of patients with ASD. Increased autoantibodies and alterations in immunoglobulins and immune cells such as T cells, B cells, monocytes, and natural killer cells are observed in individuals with ASD [126,127]. Microglial activation enhances the expression of TLRs as well as pro-inflammatory mediators. The activation of TLRs is followed by stimulation of PI3K/Akt microglial pathway which mediates increase in the production of pro-inflammatory factors accelerating neuronal damage [128]. Under these conditions, microglia attains a neurotoxic phenotype producing proteases, NO, ROS, and pro-inflammatory cytokines like TNF-α, IL-1β, IL-12, and IL-6 accelerating neuronal damage [129]. Inflammatory signaling pathways in both the central nervous system and the periphery can affect synaptic function. The effects are mediated through components including microglia, cytokines and their receptors, and major histocompatibility complex class I molecules (MHCI) [119]. Microglia and astrocytes are essential for maintaining brain homoeostasis by regulating synaptic morphology and plasticity.

Neuroinflammation appears to play a critical role in the pathogenesis of ASD. Numerous studies have shown different expressions of cytokines and chemokines in autistic patients [130]. Cytokines activate signal transduction pathways, including the JAK-STAT and PI3K/Akt/mTOR pathways, which regulate numerous cellular responses. There is emerging evidence indicating microglial activation in the brains of ASD patients. Several investigations have reported elevated plasma levels of the proinflammatory chemokine (C-C motif) ligand 5 (CCL5) in children with ASD. mTOR signaling is found to be aberrantly activated in individuals with ASD. Investigations conducted by Wang et al. showed that suppression of mTOR activity reduced the release of CCL5 from human microglia. Thus mTOR activation may play a role in regulating the expression and release of CCL5 [131]. Aberrations in Akt/mTOR signaling pathway can affect cell growth, and synthesis of cytokines in the immune system with adverse effects on the behavior [132]. Onore et al. further demonstrated that immune dysfunction in children with ASD results from irregularities in T cell signaling through the Akt/mTOR pathway. Several studies have demonstrated a vital role for microglia and astrocytes in synaptic pruning [9]. Stress and inflammation lead to the activation of the tryptophan (Trp)–kynurenine (KYN) metabolic pathway which contributes to the development of neurological and psychiatric disorders. The kynurenine pathway regulates the enzymatic conversion of tryptophan to neuroprotective product, kynurenic acid (KYNA). The TRP-KYN pathway synthesizes other metabolites including antioxidants, neurotoxins, neuroprotectants, and immunomodulators [133]. The alteration of the Trp-KYN metabolic system was also observed in patients with ASD. The mean serum levels of KYNA were significantly lower, while the ratio of KYN/KYNA was significantly elevated in the serum of children with ASD [4]. Preclinical studies have also associated maternal immune activation and microbiota profile to the complex pathogenesis of ASD [32,33].

### 4.6. Regulation of Translational Machinery in ASD

The 4E-BPs repress the translation of synaptic proteins by binding to eIF4F complex which consists of eIF4E (the cap-binding protein), eIF4A (the RNA helicase), and eIF4G (the scaffolding protein bridging RNA to ribosome) [77]. 4E-BP2, the major form of 4E-BP in the mammalian brain is phosphorylated by mTORC1 leading to its dissociation from translation initiation factor eIF4E, thus de-repressing its cap-binding activity and allowing the formation of eIF4E translation initiation complex. Autistic phenotypes are also seen in 4E-BP2 knockout and eIF4E-overexpressing mice, both being downstream effectors of mTOR regulating protein translation [134]. Omission of 4E-BP2 or overexpression of eIF4E results in augmented eIF4E complex formation, higher dendritic spine density, and behavioral irregularities suggestive of ASD. The omission of the 4E-BP2 gene specially increased the mRNA translation of synaptic protein, neuroligins. The knockdown of neuroligin was found to rescue ASD-like phenotypes in 4E-BP2 KO mice. Mutations in translation pathways in mTOR signaling cascade leads to increased protein synthesis and altered synaptic plasticity.

Genetic variation in chromosome 4q, containing the eIF4E locus, is observed in autistic patients. A rare single nucleotide polymorphism seen in ASD is linked to eIF4E gene with activating mutations in its promoter region [135]. Santini et al. have reported and described an increase in eIF4E levels in mice to be associated with enhanced cap-dependent translation and protein synthesis, resulting in an imbalance in excitatory/inhibitory transmission, unusual and repetitive behaviors suggestive of autism along with social interaction deficits. The autistic-like behaviors as well as translational defects were altered upon intracerebroventricular infusions of the cap-dependent translation inhibitor 4EGI-1 [1]. In another study by Santini et al., treatment of FXS mice with 4EGI-1 reversed the defects in cognition and spine morphology as well as normalized the phenotypes of enhanced metabotropic glutamate receptor (mGluR)-mediated LTD and dysregulated CYFIP1/eIF4E interactions in FXS mice, which further highlights the pathogenic role of exaggerated cap-dependent mRNA translation in ASD. Thus, targeting eIF4E may be an effective strategy for treating FXS [136].

## 5. Phytoconstituents in ASD

Over the years, natural products have been a source of disease-modifying agents. Several phytoconstituents exert therapeutic effects on various neurodegenerative disorders by targeting different cellular and molecular mechanisms as well as oxidative stress, inflammatory, and apoptotic pathways [137]. Plant bioactive molecules have shown beneficial pre-clinical evidence by modulating the PI3K/Akt/mTOR pathway and have been proven quite promising in ASD treatment. However, limited clinical studies have been carried out to confirm their effectiveness and associated pharmacological mechanisms in the human body.

Sharma et al. have evaluated the neuroprotective effect of chrysophanol, also known as chrysophanic acid, derived from the plant *Rheum palmatum*. Chrysophanol (10 mg/kg, 20 mg/kg, p.o.) was investigated for neuroprotective, neurochemical, and pathological variations in propionic acid-induced experimental model of autism in rats [138]. The outcomes of the study suggest that chrysophanol reinstated the altered neurochemical levels and prevented gross pathological changes as observed in autism, including demyelination volume in the rat brain. Chrysophanol was also found to improve social interaction deficits, learning, and memory in autistic rats. Furthermore, chrysophanol effectively downregulated the PI3K/Akt/mTOR pathway in autistic rats. Table 2 summarizes the various preclinical and clinical investigations on bioactive compounds from plants that exert a beneficial therapeutic role in the control and management of ASD.

Another phytoconstituent, sulforaphane, an isothiocyanate derived from vegetables such as brussels sprouts, broccoli, and cabbage, possesses antioxidant and anti-inflammatory activity [9]. Several studies have reported regulation of mTOR signaling and autophagy by sulforaphane [150,151]. An RCT in young men with moderate to severe ASD have shown that the daily administration of sulforaphane resulted in substantial improvement in social interaction, abnormal behavior, and verbal communication in the subjects without significant toxicity [152]. Another open-labeled study (ClinicalTrials.gov Identifier: NCT02654743) of sulforaphane treatment in children with ASD showed significant improvements in social responsiveness as measured by the Social Responsiveness Scale (SRS) [148]. In further investigations in children aged 3–12 years with ASD, improvements were observed in sociability and communication on the SRS (ClinicalTrials.gov Identifier: NCT02561481) [147].

Resveratrol, is a polyphenolic stilbenoid, found in berries, nuts, and grapes. It is a potent anti-neuroinflammatory agent. Resveratrol exerts neuroprotective effects by its protective effects on synaptic plasticity [9]. Several preclinical studies have established the inhibitory effect of resveratrol on mTOR [153,154]. In preclinical studies conducted in ASD rat models induced by propanoic acid, treatment with 5, 10, and 15 mg/kg resveratrol for 4 weeks has been found to restore the behavioral deficits as well as decrease inflammatory cytokines (TNF-α and IL-6) in the brain [139]. Furthermore, prenatal administration of resveratrol inhibited social impairments in a rodent model of autism induced by prenatal exposure to valproic acid [140].

The natural flavonoid, luteolin, is an mTOR inhibitor and has significant benefits in ASD [146,155]. Theoharides et al. have reported that dietary supplementation with NeuroProtek^®^, containing luteolin, quercetin, and rutin, enhanced adaptive functioning and behavioral disorders and improved inflammatory symptoms in children (aged 4–12 y) with ASD [145]. The structural analog of luteolin, methoxyluteolin, has been found to inhibit mTOR activation and consequent microglial activation and inflammation [156]. Apart from luteolin, quercetin, another mTOR inhibitor has also exhibited significant neuroprotective effects in animal models of autism [144,157].

Piperine, an alkaloid obtained from Piper longum L. and Piper nigrum L. (black pepper), possesses anxiolytic, neuroprotective, and cognition-enhancing effects [36]. Investigations conducted by Pragnya et al. have revealed improved motor deficits in ASD murine models. Piperine has ameliorative effects on behavioral alterations and oxidative stress markers [141].

De Gregorio et al. investigated the effects of 5-HT_2A_ agonist, LSD, on social behavior and glutamatergic neurotransmission in the medial PFC in male mice. Repeated LSD administration (30 μg/kg, once daily, for 7 days) was found to promote social behavior. LSD potentiates AMPA and 5-HT_2A_ synaptic responses in the mPFC. In addition, in studies of conditional KO mice deficient in Raptor (component of the mTORC1 complex) in excitatory glutamatergic neurons, the potentiation of 5-HT_2A_/AMPA synaptic responses as well as the prosocial effects of LSD were annulled, further indicating that the integrity of mTORC1 is crucial for LSD [158].

Curcumin, a polyphenol obtained from the plant Curcuma longa, possesses potent anti-inflammatory and anti-neoplastic properties. It has been found to regulate the mTOR signaling pathway and functions as an mTOR inhibitor [159,160]. Kuo et al. investigated the efficacy of curcumin in the treatment of TSC in a Tsc2 knockout mice [161]. Oral administration of solid lipid curcumin particle activates AMPK activity while inhibiting the mTOR activity in Tsc2^+/−^ mice. Curcumin further restored recognition memory loss in the mice, signifying an effective therapeutic action in TSC. In another study, administration of curcumin at a daily dose of 50, 100, and 200 mg/kg for 4 weeks in rats with propionic acid (PPA)-induced autism, improved the behavioral outcomes in addition to inhibiting oxidative-nitrosative stress, mitochondrial dysfunction, and TNF-α and MMP-9 [142].

## 6. Discussion

Multiple lines of evidence show that hyperactivation of the mTOR signaling pathway in neurons results in synaptic dysfunction, aberrant dendritic connectivity, enlarged cell size, reduced myelination, autophagic impairment, and alterations of the gene translation profile. Maintaining an optimum level of mTOR activity is necessary for neuronal cellular survival and function. The interrelationship between hyperactivation of mTOR and different syndromic forms of ASDs such as TSC, fragile X syndrome (FXS), Angelman syndrome, and Hamartoma tumor syndrome, have been proven in various clinical investigations.

**Figure 3:**
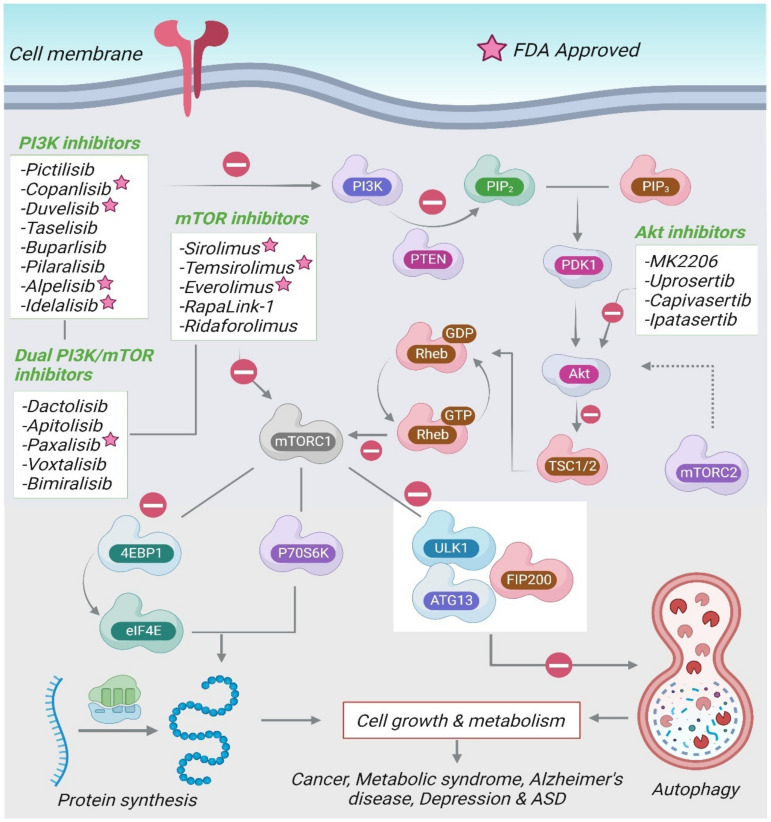
The drugs modulating the PI3K/Akt/mTOR pathway.

The etiology of ASD is poorly understood, which hinders the development of new therapeutic drug approaches. Current pharmacological treatment of ASD comprises of antidepressants, atypical antipsychotics, and anxiolytics [162]. At present, the only FDA-approved drugs for the management of ASD include the antipsychotics, risperidone and aripiprazole. However, they do not treat the core ASD symptoms and are associated with various undesirable side effects [163]. Figure 3 summarizes the various drugs modulating the PI3K/Akt/mTOR signaling pathway.

The inhibitors for the PI3K/Akt/mTOR pathway can be divided into four categories: PI3K inhibitors, mTOR inhibitors, dual PI3K/mTOR, and Akt-inhibitors. Several PI3K and Akt inhibitors have been developed as potential chemotherapeutic agents. The first generation of mTOR inhibitors, rapamycin (sirolimus) and its analogues (rapalogs), selectively prevent the activity of mTORC1 by binding to FKBP-12. mTORC1 inhibitors are the most promising drugs and are being evaluated in different subsets of ASD. The inhibitor of mTOR, everolimus, is currently under investigation in individuals with germline PTEN mutations with ASD-like behaviors.

Limited clinical studies have been carried out regarding the effects of mTORC1 inhibitors on neuropsychiatric phenotypes. The mTOR inhibitor, everolimus has been investigated for safety and possible improvements in neurocognition and behavior in individuals with PTEN gene mutation (NCT02991807) [164]. In another study, everolimus tested in children and adolescents aged 4–17 years with TSC failed to improve cognitive functioning or ASD behaviors (NCT01730209) [165]. Furthermore, in a randomized, placebo-controlled trial in children and adolescents with TSC, no noteworthy improvements in neurocognition or behavior were observed following 6 months of treatment with everolimus (NCT01289912) [163]. A randomized controlled trial using everolimus in individuals with germline PTEN mutations and comorbid ASD, to evaluate its efficacy on neurocognition and behavior is currently ongoing (ClinicalTrials.gov Identifier: NCT02991807, accessed on 2 December 2022).

Despite substantial preclinical success in animal models of TSC that support the use of mTOR inhibitors for improvement of neurocognition, clinical investigations with mTOR inhibitors have not been vastly successful. The timing of the treatment is critical regarding the course of the disease. The features of TAND manifest within the first 12–24 months of patient life. Hence, age is a key factor for mTOR-targeted treatment in TSC and other ASD phenotypes. Some adverse effects have been identified with PI3K/Akt/mTOR inhibition. A few of them are mild (e.g., hyperglycemia and hyperlipidemia), but others can be serious (e.g., hepatotoxicity, immunosuppression, stomatitis, and interstitial lung disease) [13,59]. Development of efficient pharmacological molecules, optimal dosage regimens, safety profile, and treatment duration must be established for neurocognitive and behavioral changes to clearly define their role in clinical settings. Future studies are required to target the neuropsychological domains in different subsets of ASD based on a narrower age range, symptom severity, or concurrent medications.

## 7. Conclusions and Future Perspectives

The prevalence of ASD is high, and the effective treatment is severely lacking. The identification of key signaling pathways is critical to uncover novel therapeutic targets and develop successful clinical interventions. One of the most important physiological pathways implicated in ASD is the PI3K/Akt/ mTOR signaling pathway. Collectively, several lines of evidence delineated in literature recognize the role of the PI3K/Akt/mTOR intracellular signaling pathway to play a substantial role in mediating the behavioral abnormalities that are associated with ASD. Mutations in TSC1, TSC2, and PTEN, negative regulators of mTORC1, result in ASD-like phenotypes through the disruption of the mTORC1-mediated signaling. Neuroinflammation and the progressive neuronal damage also contribute to the etiology of ASD. It is important to identify drug molecules that modulate several targets involved in the pathogenesis of ASD. Bioactive plant phytoconstituents for the treatment of ASD have been identified and these molecules need to be explored further as a therapeutic option or to provide synergistic effects, leading to reduction in dose and the adverse effects associated with a drug. Currently, mTORC1 inhibitors are the most promising drugs and are being evaluated in different subsets of ASD. With plentiful preclinical evidence, further clinical studies are needed regarding the beneficial therapeutic effect of mTORC1 inhibitors in ameliorating ASD. Pharmacotherapy combined with behavioral interventions may also be a useful treatment strategy for treating individuals with ASD.

## Figures and Tables

**Table 1:** Genes of the PI3K-Akt-mTOR pathway involved in ASD.

Human Gene	Protein	Function	Disorder
FMR1 (fragile X messenger ribonucleoprotein 1)	FMRP (fragile X mental retardation protein)	Negative regulator of protein translation	Fragile X syndrome
EIF4E	eIF4E (eukaryotic translation initiation factor 4E)	Translation initiation factor complex	ASD
PTEN	PTEN (phosphatase and tensin homolog)	Phosphatase	PHTS
TSC1/TSC2	Hamartin/tuberin	GTPase-activating protein	Tuberous sclerosis

**Table 2:** A summary of preclinical and clinical investigations of plant bioactive compounds in management of ASD.

Preclinical Investigations for Phytochemicals Effective in ASD				
Compound (s)	Method	Dose	Mechanisms and Outcomes	References
Chrysophanol	Propanoic acid-induced model of autism	(10, 20 mg/kg)	↓Akt, ↓mTOR, ↓ caspase-3, Bax, and ↑Bcl-2, ↓TNF and IL-1β, ↓AchE, ↓ LDH ↑SOD ↑GSH ↑dopamine, ↑serotonin ↑acetylcholine	[138]
Resveratrol	Propanoic acid-induced model of autism	5, 10, and 15 mg/kg	Improved behavioral, biochemical changes; reduced neuroinflammation, mitochondrial dysfunction, and oxidative/nitrosative stress; decreased inflammatory cytokines (TNF-α and IL-6)	[139]
	VPA-induced model of autism	3.6 mg/kg	Activated sirtuins and ↓ inflammation	[140]
Piperine	VPA-induced model of autism	20 mg/kg	Lowered oxidative stress; improved social behavior; ↓anxiety	[141]
Curcumin	Propanoic acid-induced model of autism	50, 100, and 200 mg/kg	Lowered oxidative-nitrosative stress, mitochondrial dysfunction, ↓TNF-α, ↓MMP-9; improved behavioral outcomes	[142]
	VPA-induced model of autism	1 g/kg	Reduced oxidative stress, ↓IL-6 levels	[143]
Quercetin	VPA-induced model of autism	50 mg/kg	Improved behavioral changes; reduced oxidative stress	[144]
Clinical studies for the therapeutic action of phytoconstituents in the management of ASD.				
Luteolin	4–14 year-old children	NeuroProtek^®^, (Luteolin: 100 mg/capsule Rutin: 30 mg/capsule Quercetin: 70 mg/capsule)	Improved behavioral outcomes; reduced gut and brain inflammation; reduced serum interleukin-6 and tumor necrosis factor	[145,146]
Sulforaphane	3–12 year-old children with ASD.	125 mg broccoli seed powder	Improvements in social responsiveness and interaction (Clinical trial identifier: NCT02561481)	[147]
	5 years to 22 years (children and young adults)	125 mg broccoli seed extract +50 mg dried broccoli sprouts	Improvement in social responsiveness (Clinical trial identifier: NCT02654743)	[148]
Epigallocatechin Gallate	18 to 55 years	400 mg/day	Improvement in memory and cognition (Clinical trial identifier: NCT01855971)	[149]

## Data Availability

This is a review and the majority of the article’s references are cited appropriately in the manuscript.

## Abbreviations

ASD	Autism Spectrum Disorder
CYFIP1	Cytoplasmic FMRP-Interacting Protein 1
eIF4E	Eukaryotic Initiation Factor 4E
4E-BP	Eukaryotic Initiation Factor 4E-Binding Protein
FMRP	Fragile X Mental Retardation Protein
FXS	Fragile X Syndrome
KO	Knockout
LTD	Long-Term Depression
LTP	Long-Term Potentiation
mGluR	Metabotropic Glutamate Receptor
mTOR	Mammalian Target of Rapamycin
mTORC1	Mammalian Target of Rapamycin Complex 1
PTEN	Phosphatase and Tensin Homolog
Rheb	Ras Homolog Enriched in Brain
S6K	Ribosomal Protein S6 Kinase
TSC	Tuberous Sclerosis Complex

## References

[1] Santini E., Huynh T.N., MacAskill A.F., Carter A.G., Pierre P., Ruggero D., Kaphzan H., Klann E. (2013). Exaggerated Translation Causes Synaptic and Behavioural Aberrations Associated with Autism. Nature.

[2] Schmunk G., Gargus J.J. (2013). Channelopathy Pathogenesis in Autism Spectrum Disorders. Front. Genet..

[3] Ellena G., Battaglia S., Làdavas E. (2020). The Spatial Effect of Fearful Faces in the Autonomic Response. Exp. Brain Res..

[4] Tanaka M., Szabó Á., Spekker E., Polyák H., Tóth F., Vécsei L. (2022). Mitochondrial Impairment: A Common Motif in Neuropsychiatric Presentation? The Link to the Tryptophan–Kynurenine Metabolic System. Cells.

[5] Borgomaneri S., Vitale F., Battaglia S., Avenanti A. (2021). Early Right Motor Cortex Response to Happy and Fearful Facial Expressions: A TMS Motor-Evoked Potential Study. Brain Sci..

[6] Varghese M., Keshav N., Jacot-Descombes S., Warda T., Wicinski B., Dickstein D.L., Harony-Nicolas H., De Rubeis S., Drapeau E., Buxbaum J.D. (2017). Autism Spectrum Disorder: Neuropathology and Animal Models. Acta Neuropathol..

[7] Khadem-Reza Z.K., Zare H. (2022). Evaluation of Brain Structure Abnormalities in Children with Autism Spectrum Disorder (ASD) Using Structural Magnetic Resonance Imaging. Egypt. J. Neurol. Psychiatry Neurosurg..

[8] Tanaka M., Spekker E., Szabó Á., Polyák H., Vécsei L. (2022). Modelling the Neurodevelopmental Pathogenesis in Neuropsychiatric Disorders. Bioactive Kynurenines and Their Analogues as Neuroprotective Agents—In Celebration of 80th Birthday of Professor Peter Riederer. J. Neural. Transm..

[9] Li Y.-J., Zhang X., Li Y.-M. (2020). Antineuroinflammatory Therapy: Potential Treatment for Autism Spectrum Disorder by Inhibiting Glial Activation and Restoring Synaptic Function. CNS Spectr..

[10] Sharma A., Hoeffer C.A., Takayasu Y., Miyawaki T., McBride S.M., Klann E., Zukin R.S. (2010). Dysregulation of MTOR Signaling in Fragile X Syndrome. J. Neurosci..

[11] Luo C., Ye W.-R., Shi W., Yin P., Chen C., He Y.-B., Chen M.-F., Zu X.-B., Cai Y. (2022). Perfect Match: MTOR Inhibitors and Tuberous Sclerosis Complex. Orphanet. J. Rare Dis..

[12] Winden K.D., Ebrahimi-Fakhari D., Sahin M. (2018). Abnormal MTOR Activation in Autism. Annu. Rev. Neurosci..

[13] Sato A., Ikeda K. (2022). Genetic and Environmental Contributions to Autism Spectrum Disorder Through Mechanistic Target of Rapamycin. Biol. Psychiatry Glob. Open Sci..

[14] Morimoto M., Hashimoto T., Tsuda Y., Nakatsu T., Kitaoka T., Kyotani S. (2020). Assessment of Oxidative Stress in Autism Spectrum Disorder Using Reactive Oxygen Metabolites and Biological Antioxidant Potential. PLoS ONE.

[15] Shuid A.N., Jayusman P.A., Shuid N., Ismail J., Kamal Nor N., Naina Mohamed I. (2020). Update on Atypicalities of Central Nervous System in Autism Spectrum Disorder. Brain Sci..

[16] Xu Z.-X., Kim G.H., Tan J.-W., Riso A.E., Sun Y., Xu E.Y., Liao G.-Y., Xu H., Lee S.-H., Do N.-Y. (2020). Elevated Protein Synthesis in Microglia Causes Autism-like Synaptic and Behavioral Aberrations. Nat. Commun..

[17] Zapata-Muñoz J., Villarejo-Zori B., Largo-Barrientos P., Boya P. (2021). Towards a Better Understanding of the Neuro-Developmental Role of Autophagy in Sickness and in Health. Cell Stress.

[18] Marotta R., Risoleo M.C., Messina G., Parisi L., Carotenuto M., Vetri L., Roccella M. (2020). The Neurochemistry of Autism. Brain Sci..

[19] Garro-Martínez E., Fullana M.N., Florensa-Zanuy E., Senserrich J., Paz V., Ruiz-Bronchal E., Adell A., Castro E., Díaz Á., Pazos Á. (2021). MTOR Knockdown in the Infralimbic Cortex Evokes A Depressive-like State in Mouse. Int. J. Mol. Sci..

[20] Singla R., Mishra A., Cao R. (2022). The Trilateral Interactions between Mammalian Target of Rapamycin (MTOR) Signaling, the Circadian Clock, and Psychiatric Disorders: An Emerging Model. Transl. Psychiatry.

[21] Birdsall V., Waites C.L. (2019). Autophagy at the Synapse. Neurosci. Lett..

[22] Crino P.B. (2016). The MTOR Signalling Cascade: Paving New Roads to Cure Neurological Disease. Nat. Rev. Neurol..

[23] Deng Z., Zhou X., Lu J.-H., Yue Z. (2021). Autophagy Deficiency in Neurodevelopmental Disorders. Cell Biosci..

[24] Rapaka D., Bitra V.R., Challa S.R., Adiukwu P.C. (2022). MTOR Signaling as a Molecular Target for the Alleviation of Alzheimer’s Disease Pathogenesis. Neurochem. Int..

[25] Zhu Z., Yang C., Iyaswamy A., Krishnamoorthi S., Sreenivasmurthy S.G., Liu J., Wang Z., Tong B.C.-K., Song J., Lu J. (2019). Balancing MTOR Signaling and Autophagy in the Treatment of Parkinson’s Disease. Int. J. Mol. Sci..

[26] Sadowski K., Kotulska-Jóźwiak K., Jóźwiak S. (2015). Role of MTOR Inhibitors in Epilepsy Treatment. Pharmacol. Rep..

[27] Athira K.V., Mohan A.S., Chakravarty S. (2020). Rapid Acting Antidepressants in the MTOR Pathway: Current Evidence. Brain Res. Bull..

[28] Gao R., Penzes P. (2015). Common Mechanisms of Excitatory and Inhibitory Imbalance in Schizophrenia and Autism Spectrum Disorders. Curr. Mol. Med..

[29] Machado-Vieira R., Zanetti M.V., Teixeira A.L., Uno M., Valiengo L.L., Soeiro-de-Souza M.G., Oba-Shinjo S.M., de Sousa R.T., Zarate C.A., Gattaz W.F. (2015). Decreased AKT1/MTOR Pathway MRNA Expression in Short-Term Bipolar Disorder. Eur. Neuropsychopharmacol..

[30] Sato A., Kotajima-Murakami H., Tanaka M., Katoh Y., Ikeda K. (2022). Influence of Prenatal Drug Exposure, Maternal Inflammation, and Parental Aging on the Development of Autism Spectrum Disorder. Front. Psychiatry.

[31] Rosenfeld C.S. (2015). Microbiome Disturbances and Autism Spectrum Disorders. Drug Metab. Dispos..

[32] Lee G.A., Lin Y.-K., Lai J.-H., Lo Y.-C., Yang Y.-C.S.H., Ye S.-Y., Lee C.-J., Wang C.-C., Chiang Y.-H., Tseng S.-H. (2021). Maternal Immune Activation Causes Social Behavior Deficits and Hypomyelination in Male Rat Offspring with an Autism-Like Microbiota Profile. Brain Sci..

[33] Abuaish S., Al-Otaibi N.M., Abujamel T.S., Alzahrani S.A., Alotaibi S.M., AlShawakir Y.A., Aabed K., El-Ansary A. (2021). Fecal Transplant and Bifidobacterium Treatments Modulate Gut Clostridium Bacteria and Rescue Social Impairment and Hippocampal BDNF Expression in a Rodent Model of Autism. Brain Sci..

[34] Fang K., Liu D., Pathak S.S., Yang B., Li J., Karthikeyan R., Chao O.Y., Yang Y.-M., Jin V.X., Cao R. (2021). Disruption of Circadian Rhythms by Ambient Light during Neurodevelopment Leads to Autistic-like Molecular and Behavioral Alterations in Adult Mice. Cells.

[35] Hussain H., Ahmad S., Shah S.W.A., Ullah A., Almehmadi M., Abdulaziz O., Allahyani M., Alsaiari A.A., Halawi M., Alamer E. (2022). Investigation of Antistress and Antidepressant Activities of Synthetic Curcumin Analogues: Behavioral and Biomarker Approach. Biomedicines.

[36] Cruz-Martins N., Quispe C., Kırkın C., Şenol E., Zuluğ A., Özçelik B., Ademiluyi A.O., Oyeniran O.H., Semwal P., Kumar M. (2021). Paving Plant-Food-Derived Bioactives as Effective Therapeutic Agents in Autism Spectrum Disorder. Oxid Med. Cell Longev..

[37] Latacz A., Russell J.A., Oc E., Zubel J., Pierzcha K. (2015). Review MTOR Pathway–Novel Modulator of Astrocyte Activity. Folia Biol..

[38] Ryskalin L., Limanaqi F., Frati A., Busceti C., Fornai F. (2018). MTOR-Related Brain Dysfunctions in Neuropsychiatric Disorders. Int. J. Mol. Sci..

[39] Cai Z., Yan L.-J. (2013). Rapamycin, Autophagy, and Alzheimer’s Disease. J. Biochem. Pharmacol. Res..

[40] Cai Z., Zhao B., Li K., Zhang L., Li C., Quazi S.H., Tan Y. (2012). Mammalian Target of Rapamycin: A Valid Therapeutic Target through the Autophagy Pathway for Alzheimer’s Disease?. J. Neurosci. Res..

[41] Friedman L.G., Qureshi Y.H., Yu W.H. (2015). Promoting Autophagic Clearance: Viable Therapeutic Targets in Alzheimer’s Disease. Neurotherapeutics.

[42] Maiese K. (2017). Moving to the Rhythm with Clock (Circadian) Genes, Autophagy, MTOR, and SIRT1 in Degenerative Disease and Cancer. Curr. Neurovascular Res..

[43] Maiese K. (2014). Taking Aim at Alzheimer’s Disease through the Mammalian Target of Rapamycin. Ann. Med..

[44] Bockaert J., Marin P. (2015). MTOR in Brain Physiology and Pathologies. Physiol. Rev..

[45] Kim Y.C., Guan K.-L. (2015). MTOR: A Pharmacologic Target for Autophagy Regulation. J. Clin. Investig..

[46] Lee H.-K., Kwon B., Lemere C.A., de la Monte S., Itamura K., Ha A.Y., Querfurth H.W. (2017). MTORC2 (Rictor) in Alzheimer’s Disease and Reversal of Amyloid-β Expression-Induced Insulin Resistance and Toxicity in Rat Primary Cortical Neurons. J. Alzheimer’s Dis..

[47] Kuang H., Tan C., Tian H., Liu L., Yang M., Hong F., Yang S. (2019). Exploring the Bi-directional Relationship between Autophagy and Alzheimer’s Disease. CNS Neurosci. Ther..

[48] Cai Z., Zhou Y., Xiao M., Yan L.-J., He W. (2015). Activation of MTOR: A Culprit of Alzheimer&rsquo;s Disease?. Neuropsychiatr. Dis. Treat..

[49] Yang H., Rudge D.G., Koos J.D., Vaidialingam B., Yang H.J., Pavletich N.P. (2013). MTOR Kinase Structure, Mechanism and Regulation. Nature.

[50] Galvan V., Hart M.J. (2016). Vascular MTOR-Dependent Mechanisms Linking the Control of Aging to Alzheimer’s Disease. Biochim. Biophys. Acta Mol. Basis Dis..

[51] Singh A.K., Kashyap M.P., Tripathi V.K., Singh S., Garg G., Rizvi S.I. (2017). Neuroprotection Through Rapamycin-Induced Activation of Autophagy and PI3K/Akt1/MTOR/CREB Signaling Against Amyloid-β-Induced Oxidative Stress, Synaptic/Neurotransmission Dysfunction, and Neurodegeneration in Adult Rats. Mol. Neurobiol..

[52] Guo J., Cheng J., North B.J., Wei W. (2017). Functional Analyses of Major Cancer-Related Signaling Pathways in Alzheimer’s Disease Etiology. Biochim. Biophys. Acta Rev. Cancer.

[53] Perluigi M., Di Domenico F., Barone E., Butterfield D.A. (2021). MTOR in Alzheimer Disease and Its Earlier Stages: Links to Oxidative Damage in the Progression of This Dementing Disorder. Free. Radic. Biol. Med..

[54] Kou X., Chen D., Chen N. (2019). Physical Activity Alleviates Cognitive Dysfunction of Alzheimer’s Disease through Regulating the MTOR Signaling Pathway. Int. J. Mol. Sci..

[55] Cai Z., Yan L.-J., Li K., Quazi S.H., Zhao B. (2012). Roles of AMP-Activated Protein Kinase in Alzheimer’s Disease. Neuromol. Med..

[56] Maiese K. (2014). Driving Neural Regeneration through the Mammalian Target of Rapamycin. Neural. Regen Res..

[57] Weinberg M.A. (2016). RES-529: A PI3K/AKT/MTOR Pathway Inhibitor That Dissociates the MTORC1 and MTORC2 Complexes. Anti-Cancer Drugs.

[58] Pourtalebi Jahromi L., Sasanipour Z., Azadi A. (2018). Promising Horizon to Alleviate Alzeheimer’s Disease Pathological Hallmarks via Inhibiting MTOR Signaling Pathway: A New Application for a Commonplace Analgesic. Med. Hypotheses.

[59] Popova N.V., Jücker M. (2021). The Role of MTOR Signaling as a Therapeutic Target in Cancer. Int. J. Mol. Sci..

[60] Filomeni G., De Zio D., Cecconi F. (2015). Oxidative Stress and Autophagy: The Clash between Damage and Metabolic Needs. Cell Death Differ..

[61] Chandran A., Iyo A.H., Jernigan C.S., Legutko B., Austin M.C., Karolewicz B. (2013). Reduced Phosphorylation of the MTOR Signaling Pathway Components in the Amygdala of Rats Exposed to Chronic Stress. Prog. Neuro-Psychopharmacol. Biol. Psychiatry.

[62] Cordero J.G., García-Escudero R., Avila J., Gargini R., García-Escudero V. (2018). Benefit of Oleuropein Aglycone for Alzheimer’s Disease by Promoting Autophagy. Oxidative Med. Cell. Longev..

[63] Eshraghi M., Ahmadi M., Afshar S., Lorzadeh S., Adlimoghaddam A., Rezvani Jalal N., West R., Dastghaib S., Igder S., Torshizi S.R.N. (2022). Enhancing Autophagy in Alzheimer’s Disease through Drug Repositioning. Pharmacol. Ther..

[64] Festa B.P., Barbosa A.D., Rob M., Rubinsztein D.C. (2021). The Pleiotropic Roles of Autophagy in Alzheimer’s Disease: From Pathophysiology to Therapy. Curr. Opin. Pharmacol..

[65] Mitjans M., Begemann M., Ju A., Dere E., Wüstefeld L., Hofer S., Hassouna I., Balkenhol J., Oliveira B., van der Auwera S. (2017). Sexual Dimorphism of AMBRA1-Related Autistic Features in Human and Mouse. Transl. Psychiatry.

[66] Chen J. (2014). Dysregulation of the IGF-I/PI3K/AKT/MTOR Signaling Pathway in Autism Spectrum Disorders. Int. J. Dev. Neurosci..

[67] Chaudry S., Vasudevan N. (2022). MTOR-Dependent Spine Dynamics in Autism. Front. Mol. Neurosci..

[68] Kassai H., Sugaya Y., Noda S., Nakao K., Maeda T., Kano M., Aiba A. (2014). Selective Activation of MTORC1 Signaling Recapitulates Microcephaly, Tuberous Sclerosis, and Neurodegenerative Diseases. Cell Rep..

[69] Opazo P., Watabe A.M., Grant S.G.N., O’Dell T.J. (2003). Phosphatidylinositol 3-Kinase Regulates the Induction of Long-Term Potentiation through Extracellular Signal-Related Kinase-Independent Mechanisms. J. Neurosci..

[70] Zhang J., Zhang J.-X., Zhang Q.-L. (2016). PI3K/AKT/MTOR-Mediated Autophagy in the Development of Autism Spectrum Disorder. Brain Res. Bull..

[71] Lieberman O.J., Cartocci V., Pigulevskiy I., Molinari M., Carbonell J., Broseta M.B., Post M.R., Sulzer D., Borgkvist A., Santini E. (2020). MTOR Suppresses Macroautophagy During Striatal Postnatal Development and Is Hyperactive in Mouse Models of Autism Spectrum Disorders. Front. Cell Neurosci..

[72] Onore C., Yang H., Van de Water J., Ashwood P. (2017). Dynamic Akt/MTOR Signaling in Children with Autism Spectrum Disorder. Front. Pediatr..

[73] Rosina E., Battan B., Siracusano M., Di Criscio L., Hollis F., Pacini L., Curatolo P., Bagni C. (2019). Disruption of MTOR and MAPK Pathways Correlates with Severity in Idiopathic Autism. Transl. Psychiatry.

[74] Wang F., Wang L., Xiong Y., Deng J., Lü M., Tang B., Zhang X., Li Y. (2022). [Mechanism of valproic acid-induced dendritic spine and synaptic impairment in the prefrontal cortex for causing core autistic symptoms in mice. Nan Fang Yi Ke Da Xue Xue Bao.

[75] Hagerman R.J., Berry-Kravis E., Hazlett H.C., Bailey D.B., Moine H., Kooy R.F., Tassone F., Gantois I., Sonenberg N., Mandel J.L. (2017). Fragile X Syndrome. Nat. Rev. Dis. Primers.

[76] Casingal C.R., Kikkawa T., Inada H., Sasaki Y., Osumi N. (2020). Identification of FMRP Target MRNAs in the Developmental Brain: FMRP Might Coordinate Ras/MAPK, Wnt/β-Catenin, and MTOR Signaling during Corticogenesis. Mol. Brain.

[77] Sato A. (2016). MTOR, a Potential Target to Treat Autism Spectrum Disorder. CNS Neurol. Disord. Drug Targets.

[78] Kazdoba T.M., Leach P.T., Silverman J.L., Crawley J.N. (2014). Modeling Fragile X Syndrome in the Fmr1 Knockout Mouse. Intractable Rare Dis. Res..

[79] Grossman A.W., Elisseou N.M., McKinney B.C., Greenough W.T. (2006). Hippocampal Pyramidal Cells in Adult Fmr1 Knockout Mice Exhibit an Immature-Appearing Profile of Dendritic Spines. Brain Res..

[80] Nisar S., Bhat A.A., Masoodi T., Hashem S., Akhtar S., Ali T.A., Amjad S., Chawla S., Bagga P., Frenneaux M.P. (2022). Genetics of Glutamate and Its Receptors in Autism Spectrum Disorder. Mol. Psychiatry.

[81] Huber K.M., Gallagher S.M., Warren S.T., Bear M.F. (2002). Altered Synaptic Plasticity in a Mouse Model of Fragile X Mental Retardation. Proc. Natl. Acad. Sci. USA.

[82] Thomas A.M., Bui N., Perkins J.R., Yuva-Paylor L.A., Paylor R. (2012). Group I Metabotropic Glutamate Receptor Antagonists Alter Select Behaviors in a Mouse Model for Fragile X Syndrome. Psychopharmacology.

[83] Saré R.M., Song A., Loutaev I., Cook A., Maita I., Lemons A., Sheeler C., Smith C.B. (2018). Negative Effects of Chronic Rapamycin Treatment on Behavior in a Mouse Model of Fragile X Syndrome. Front. Mol. Neurosci..

[84] Hoeffer C.A., Sanchez E., Hagerman R.J., Mu Y., Nguyen D.V., Wong H., Whelan A.M., Zukin R.S., Klann E., Tassone F. (2012). Altered MTOR Signaling and Enhanced CYFIP2 Expression Levels in Subjects with Fragile X Syndrome. Genes Brain Behav..

[85] Gross C., Nakamoto M., Yao X., Chan C.-B., Yim S.Y., Ye K., Warren S.T., Bassell G.J. (2010). Excess Phosphoinositide 3-Kinase Subunit Synthesis and Activity as a Novel Therapeutic Target in Fragile X Syndrome. J. Neurosci..

[86] Gantois I., Khoutorsky A., Popic J., Aguilar-Valles A., Freemantle E., Cao R., Sharma V., Pooters T., Nagpal A., Skalecka A. (2017). Metformin Ameliorates Core Deficits in a Mouse Model of Fragile X Syndrome. Nat. Med..

[87] Qin M., Kang J., Burlin T.V., Jiang C., Smith C.B. (2005). Postadolescent Changes in Regional Cerebral Protein Synthesis: An In Vivo Study in the Fmr1 Null Mouse. J. Neurosci..

[88] Napoli I., Mercaldo V., Boyl P.P., Eleuteri B., Zalfa F., De Rubeis S., Di Marino D., Mohr E., Massimi M., Falconi M. (2008). The Fragile X Syndrome Protein Represses Activity-Dependent Translation through CYFIP1, a New 4E-BP. Cell.

[89] Bhattacharya A., Kaphzan H., Alvarez-Dieppa A.C., Murphy J.P., Pierre P., Klann E. (2012). Genetic Removal of P70 S6 Kinase 1 Corrects Molecular, Synaptic, and Behavioral Phenotypes in Fragile X Syndrome Mice. Neuron.

[90] Yan J., Porch M.W., Court-Vazquez B., Bennett M.V.L., Zukin R.S. (2018). Activation of Autophagy Rescues Synaptic and Cognitive Deficits in Fragile X Mice. Proc. Natl. Acad. Sci. USA.

[91] Lugo J.N., Smith G.D., Arbuckle E.P., White J., Holley A.J., Floruta C.M., Ahmed N., Gomez M.C., Okonkwo O. (2014). Deletion of PTEN Produces Autism-like Behavioral Deficits and Alterations in Synaptic Proteins. Front. Mol. Neurosci..

[92] Zhou J., Parada L.F. (2012). PTEN Signaling in Autism Spectrum Disorders. Curr. Opin. Neurobiol..

[93] Kwon C.-H., Luikart B.W., Powell C.M., Zhou J., Matheny S.A., Zhang W., Li Y., Baker S.J., Parada L.F. (2006). Pten Regulates Neuronal Arborization and Social Interaction in Mice. Neuron.

[94] Yeung K.S., Tso W.W.Y., Ip J.J.K., Mak C.C.Y., Leung G.K.C., Tsang M.H.Y., Ying D., Pei S.L.C., Lee S.L., Yang W. (2017). Identification of Mutations in the PI3K-AKT-MTOR Signalling Pathway in Patients with Macrocephaly and Developmental Delay and/or Autism. Mol. Autism..

[95] Busch R.M., Srivastava S., Hogue O., Frazier T.W., Klaas P., Hardan A., Martinez-Agosto J.A., Sahin M., Eng C. (2019). Neurobehavioral Phenotype of Autism Spectrum Disorder Associated with Germline Heterozygous Mutations in PTEN. Transl. Psychiatry.

[96] Clipperton-Allen A.E., Page D.T. (2014). Pten Haploinsufficient Mice Show Broad Brain Overgrowth but Selective Impairments in Autism-Relevant Behavioral Tests. Hum. Mol. Genet..

[97] Tai C., Chang C.-W., Yu G.-Q., Lopez I., Yu X., Wang X., Guo W., Mucke L. (2020). Tau Reduction Prevents Key Features of Autism in Mouse Models. Neuron.

[98] Zhou J., Blundell J., Ogawa S., Kwon C.-H., Zhang W., Sinton C., Powell C.M., Parada L.F. (2009). Pharmacological Inhibition of MTORC1 Suppresses Anatomical, Cellular, and Behavioral Abnormalities in Neural-Specific Pten Knock-Out Mice. J. Neurosci..

[99] Varga E.A., Pastore M., Prior T., Herman G.E., McBride K.L. (2009). The Prevalence of PTEN Mutations in a Clinical Pediatric Cohort with Autism Spectrum Disorders, Developmental Delay, and Macrocephaly. Genet. Med..

[100] Skelton P.D., Stan R.V., Luikart B.W. (2019). The Role of PTEN in Neurodevelopment. Complex Psychiatry.

[101] Frazier T.W., Embacher R., Tilot A.K., Koenig K., Mester J., Eng C. (2015). Molecular and Phenotypic Abnormalities in Individuals with Germline Heterozygous PTEN Mutations and Autism. Mol. Psychiatry.

[102] Kuo H.-Y., Liu F.-C. (2018). Molecular Pathology and Pharmacological Treatment of Autism Spectrum Disorder-Like Phenotypes Using Rodent Models. Front. Cell Neurosci..

[103] Xu J., Du Y., Xu J., Hu X., Gu L., Li X., Hu P., Liao T., Xia Q., Sun Q. (2019). Neuroligin 3 Regulates Dendritic Outgrowth by Modulating Akt/MTOR Signaling. Front. Cell Neurosci..

[104] Potter W.B., Basu T., O’Riordan K.J., Kirchner A., Rutecki P., Burger C., Roopra A. (2013). Reduced Juvenile Long-Term Depression in Tuberous Sclerosis Complex Is Mitigated in Adults by Compensatory Recruitment of MGluR5 and Erk Signaling. PLoS Biol..

[105] Curatolo P., Moavero R. (2012). MTOR Inhibitors in Tuberous Sclerosis Complex. Curr. Neuropharmacol..

[106] Lee K.-M., Hwang S.-K., Lee J.-A. (2013). Neuronal Autophagy and Neurodevelopmental Disorders. Exp. Neurobiol..

[107] Caglayan A.O. (2010). Genetic Causes of Syndromic and Non-Syndromic Autism. Dev. Med. Child Neurol..

[108] Goorden S.M.I., van Woerden G.M., van der Weerd L., Cheadle J.P., Elgersma Y. (2007). Cognitive Deficits in Tsc1^+^/^−^ Mice in the Absence of Cerebral Lesions and Seizures. Ann. Neurol..

[109] Tsai P.T., Hull C., Chu Y., Greene-Colozzi E., Sadowski A.R., Leech J.M., Steinberg J., Crawley J.N., Regehr W.G., Sahin M. (2012). Autistic-like Behavior and Cerebellar Dysfunction in Purkinje Cell Tsc1 Mutant Mice. Nature.

[110] Sato A., Kasai S., Kobayashi T., Takamatsu Y., Hino O., Ikeda K., Mizuguchi M. (2012). Rapamycin Reverses Impaired Social Interaction in Mouse Models of Tuberous Sclerosis Complex. Nat. Commun..

[111] Tang G., Gudsnuk K., Kuo S.-H., Cotrina M.L., Rosoklija G., Sosunov A., Sonders M.S., Kanter E., Castagna C., Yamamoto A. (2014). Loss of MTOR-Dependent Macroautophagy Causes Autistic-like Synaptic Pruning Deficits. Neuron.

[112] Reith R.M., McKenna J., Wu H., Hashmi S.S., Cho S.-H., Dash P.K., Gambello M.J. (2013). Loss of Tsc2 in Purkinje Cells Is Associated with Autistic-like Behavior in a Mouse Model of Tuberous Sclerosis Complex. Neurobiol. Dis..

[113] Pagani M., Barsotti N., Bertero A., Trakoshis S., Ulysse L., Locarno A., Miseviciute I., De Felice A., Canella C., Supekar K. (2021). MTOR-Related Synaptic Pathology Causes Autism Spectrum Disorder-Associated Functional Hyperconnectivity. Nat. Commun..

[114] Martin P., Wagh V., Reis S.A., Erdin S., Beauchamp R.L., Shaikh G., Talkowski M., Thiele E., Sheridan S.D., Haggarty S.J. (2020). TSC Patient-Derived Isogenic Neural Progenitor Cells Reveal Altered Early Neurodevelopmental Phenotypes and Rapamycin-Induced MNK-EIF4E Signaling. Mol. Autism..

[115] Ehninger D., Silva A.J. (2011). Rapamycin for Treating Tuberous Sclerosis and Autism Spectrum Disorders. Trends Mol. Med..

[116] Hui K.K., Tanaka M. (2019). Autophagy Links MTOR and GABA Signaling in the Brain. Autophagy.

[117] Antoine M.W., Langberg T., Schnepel P., Feldman D.E. (2019). Increased Excitation-Inhibition Ratio Stabilizes Synapse and Circuit Excitability in Four Autism Mouse Models. Neuron.

[118] Nelson S.B., Valakh V. (2015). Excitatory/Inhibitory Balance and Circuit Homeostasis in Autism Spectrum Disorders. Neuron.

[119] Jiang C.-C., Lin L.-S., Long S., Ke X.-Y., Fukunaga K., Lu Y.-M., Han F. (2022). Signalling Pathways in Autism Spectrum Disorder: Mechanisms and Therapeutic Implications. Sig. Transduct. Target Ther..

[120] Reichelt A.C., Rodgers R.J., Clapcote S.J. (2012). The Role of Neurexins in Schizophrenia and Autistic Spectrum Disorder. Neuropharmacology.

[121] Mehta M.V., Gandal M.J., Siegel S.J. (2011). MGluR5-Antagonist Mediated Reversal of Elevated Stereotyped, Repetitive Behaviors in the VPA Model of Autism. PLoS ONE.

[122] Sun Y.-Y., Chen W.-J., Huang Z.-P., Yang G., Wu M.-L., Xu D.-E., Yang W.-L., Luo Y.-C., Xiao Z.-C., Xu R.-X. (2022). TRIM32 Deficiency Impairs the Generation of Pyramidal Neurons in Developing Cerebral Cortex. Cells.

[123] Lionel A.C., Tammimies K., Vaags A.K., Rosenfeld J.A., Ahn J.W., Merico D., Noor A., Runke C.K., Pillalamarri V.K., Carter M.T. (2014). Disruption of the ASTN2/TRIM32 Locus at 9q33.1 Is a Risk Factor in Males for Autism Spectrum Disorders, ADHD and Other Neurodevelopmental Phenotypes. Hum. Mol. Genet..

[124] Zhu J.-W., Zou M.-M., Li Y.-F., Chen W.-J., Liu J.-C., Chen H., Fang L.-P., Zhang Y., Wang Z.-T., Chen J.-B. (2020). Absence of TRIM32 Leads to Reduced GABAergic Interneuron Generation and Autism-like Behaviors in Mice via Suppressing MTOR Signaling. Cerebral. Cortex.

[125] Hui K., Takashima N., Watanabe A., Chater T., Matsukawa H., Nekooki-Machida Y., Nilsson P., Endo R., Goda Y., Saido T. (2019). GABARAPs Dysfunction by Autophagy Deficiency in Adolescent Brain Impairs GABA A Receptor Trafficking and Social Behavior. Sci. Adv..

[126] Bjorklund G., Saad K., Chirumbolo S., Kern J.K., Geier D.A., Geier M.R., Urbina M.A. (2016). Immune Dysfunction and Neuroinflammation in Autism Spectrum Disorder. Acta Neurobiol. Exp..

[127] Theoharides T.C., Tsilioni I., Patel A.B., Doyle R. (2016). Atopic Diseases and Inflammation of the Brain in the Pathogenesis of Autism Spectrum Disorders. Transl. Psychiatry.

[128] Cianciulli A., Porro C., Calvello R., Trotta T., Lofrumento D.D., Panaro M.A. (2020). Microglia Mediated Neuroinflammation: Focus on PI3K Modulation. Biomolecules.

[129] London A., Cohen M., Schwartz M. (2013). Microglia and Monocyte-Derived Macrophages: Functionally Distinct Populations That Act in Concert in CNS Plasticity and Repair. Front. Cell Neurosci..

[130] Matta S.M., Hill-Yardin E.L., Crack P.J. (2019). The Influence of Neuroinflammation in Autism Spectrum Disorder. Brain Behav. Immun..

[131] Wang B., Qin Y., Wu Q., Li X., Xie D., Zhao Z., Duan S. (2022). MTOR Signaling Pathway Regulates the Release of Proinflammatory Molecule CCL5 Implicated in the Pathogenesis of Autism Spectrum Disorder. Front. Immunol..

[132] Onore C., Careaga M., Ashwood P. (2012). The Role of Immune Dysfunction in the Pathophysiology of Autism. Brain Behav. Immun..

[133] Tanaka M., Tóth F., Polyák H., Szabó Á., Mándi Y., Vécsei L. (2021). Immune Influencers in Action: Metabolites and Enzymes of the Tryptophan-Kynurenine Metabolic Pathway. Biomedicines.

[134] Gkogkas C.G., Khoutorsky A., Ran I., Rampakakis E., Nevarko T., Weatherill D.B., Vasuta C., Yee S., Truitt M., Dallaire P. (2013). Autism-Related Deficits via Dysregulated EIF4E-Dependent Translational Control. Nature.

[135] Neves-Pereira M., Müller B., Massie D., Williams J.H.G., O’Brien P.C.M., Hughes A., Shen S.-B., Clair D.S., Miedzybrodzka Z. (2009). Deregulation of EIF4E: A Novel Mechanism for Autism. J. Med. Genet..

[136] Santini E., Huynh T.N., Longo F., Koo S.Y., Mojica E., D’Andrea L., Bagni C., Klann E. (2017). Reducing EIF4E-EIF4G Interactions Restores the Balance between Protein Synthesis and Actin Dynamics in Fragile X Syndrome Model Mice. Sci. Signal..

[137] Fakhri S. (2021). Natural Products Attenuate PI3K/Akt/MTOR Signaling Pathway: A Promising Strategy in Regulating Neurodegeneration. Phytomedicine.

[138] Sharma A., Bhalla S., Mehan S. (2022). PI3K/AKT/MTOR Signalling Inhibitor Chrysophanol Ameliorates Neurobehavioural and Neurochemical Defects in Propionic Acid-Induced Experimental Model of Autism in Adult Rats. Metab. Brain Dis..

[139] Bhandari R., Kuhad A. (2017). Resveratrol Suppresses Neuroinflammation in the Experimental Paradigm of Autism Spectrum Disorders. Neurochem. Int..

[140] Bambini-Junior V., Zanatta G., Della Flora Nunes G., Mueller de Melo G., Michels M., Fontes-Dutra M., Nogueira Freire V., Riesgo R., Gottfried C. (2014). Resveratrol Prevents Social Deficits in Animal Model of Autism Induced by Valproic Acid. Neurosci. Lett..

[141] Pragnya B., Kameshwari J.S.L., Veeresh B. (2014). Ameliorating Effect of Piperine on Behavioral Abnormalities and Oxidative Markers in Sodium Valproate Induced Autism in BALB/C Mice. Behav. Brain Res..

[142] Bhandari R., Kuhad A. (2015). Neuropsychopharmacotherapeutic Efficacy of Curcumin in Experimental Paradigm of Autism Spectrum Disorders. Life Sci..

[143] Al-Askar M., Bhat R.S., Selim M., Al-Ayadhi L., El-Ansary A. (2017). Postnatal Treatment Using Curcumin Supplements to Amend the Damage in VPA-Induced Rodent Models of Autism. BMC Complement. Altern. Med..

[144] de Mattos B.D.S., Soares M.S.P., Spohr L., Pedra N.S., Teixeira F.C., de Souza A.A., Stefanello F.M., Baldissarelli J., Gamaro G.D., Spanevello R.M. (2020). Quercetin Prevents Alterations of Behavioral Parameters, Delta-Aminolevulinic Dehydratase Activity, and Oxidative Damage in Brain of Rats in a Prenatal Model of Autism. Int. J. Dev. Neurosci..

[145] Theoharides T.C., Asadi S., Panagiotidou S. (2012). A Case Series of a Luteolin Formulation (NeuroProtek^®^) in Children with Autism Spectrum Disorders. Int. J. Immunopathol. Pharmacol..

[146] Tsilioni I., Taliou A., Francis K., Theoharides T.C. (2015). Children with Autism Spectrum Disorders, Who Improved with a Luteolin-Containing Dietary Formulation, Show Reduced Serum Levels of TNF and IL-6. Transl. Psychiatry.

[147] Zimmerman A.W., Singh K., Connors S.L., Liu H., Panjwani A.A., Lee L.-C., Diggins E., Foley A., Melnyk S., Singh I.N. (2021). Randomized Controlled Trial of Sulforaphane and Metabolite Discovery in Children with Autism Spectrum Disorder. Mol. Autism..

[148] Bent S., Lawton B., Warren T., Widjaja F., Dang K., Fahey J.W., Cornblatt B., Kinchen J.M., Delucchi K., Hendren R.L. (2018). Identification of Urinary Metabolites That Correlate with Clinical Improvements in Children with Autism Treated with Sulforaphane from Broccoli. Mol. Autism..

[149] Van Aller G.S., Carson J.D., Tang W., Peng H., Zhao L., Copeland R.A., Tummino P.J., Luo L. (2011). Epigallocatechin Gallate (EGCG), a Major Component of Green Tea, Is a Dual Phosphoinositide-3-Kinase/MTOR Inhibitor. Biochem. Biophys. Res. Commun..

[150] Pawlik A., Wiczk A., Kaczyńska A., Antosiewicz J., Herman-Antosiewicz A. (2013). Sulforaphane Inhibits Growth of Phenotypically Different Breast Cancer Cells. Eur. J. Nutr..

[151] Zhang Y., Gilmour A., Ahn Y.-H., de la Vega L., Dinkova-Kostova A.T. (2021). The Isothiocyanate Sulforaphane Inhibits MTOR in an NRF2-Independent Manner. Phytomedicine.

[152] Singh K., Connors S.L., Macklin E.A., Smith K.D., Fahey J.W., Talalay P., Zimmerman A.W. (2014). Sulforaphane Treatment of Autism Spectrum Disorder (ASD). Proc. Natl. Acad. Sci. USA.

[153] Park D., Jeong H., Lee M.N., Koh A., Kwon O., Yang Y.R., Noh J., Suh P.-G., Park H., Ryu S.H. (2016). Resveratrol Induces Autophagy by Directly Inhibiting MTOR through ATP Competition. Sci. Rep..

[154] Tian Y., Song W., Li D., Cai L., Zhao Y. (2019). Resveratrol As A Natural Regulator Of Autophagy For Prevention And Treatment Of Cancer. Onco. Targets Ther..

[155] Liu Y., Huang J., Zheng X., Yang X., Ding Y., Fang T., Zhang Y., Wang S., Zhang X., Luo X. (2017). Luteolin, a Natural Flavonoid, Inhibits Methylglyoxal Induced Apoptosis via the MTOR/4E-BP1 Signaling Pathway. Sci. Rep..

[156] Patel A.B., Tsilioni I., Leeman S.E., Theoharides T.C. (2016). Neurotensin Stimulates Sortilin and MTOR in Human Microglia Inhibitable by Methoxyluteolin, a Potential Therapeutic Target for Autism. Proc. Natl. Acad. Sci. USA.

[157] Bruning A. (2013). Inhibition of MTOR Signaling by Quercetin in Cancer Treatment and Prevention. Anti-Cancer Agents Med. Chem. Anti-Cancer Agents.

[158] De Gregorio D., Popic J., Enns J.P., Inserra A., Skalecka A., Markopoulos A., Posa L., Lopez-Canul M., Qianzi H., Lafferty C.K. (2021). Lysergic Acid Diethylamide (LSD) Promotes Social Behavior through MTORC1 in the Excitatory Neurotransmission. Proc. Natl. Acad. Sci. USA.

[159] Yu S., Shen G., Khor T.O., Kim J.-H., Kong A.-N.T. (2008). Curcumin Inhibits Akt/Mammalian Target of Rapamycin Signaling through Protein Phosphatase-Dependent Mechanism. Mol. Cancer Ther..

[160] Johnson S.M., Gulhati P., Arrieta I., Wang X., Uchida T., Gao T., Evers B.M. (2009). Curcumin Inhibits Proliferation of Colorectal Carcinoma by Modulating Akt/MTOR Signaling. Anticancer. Res..

[161] Kuo C.-J. (2019). Potential Therapeutic Effect of Curcumin, a Natural MTOR Inhibitor, in Tuberous Sclerosis Complex. Phytomedicine.

[162] Robinson-Agramonte M.D.L.A., Michalski B., Vidal-Martinez B., Hernández L.R., Santiesteban M.W., Fahnestock M. (2022). BDNF, ProBDNF and IGF-1 Serum Levels in Naïve and Medicated Subjects with Autism. Sci. Rep..

[163] DeSpenza T., Carlson M., Panchagnula S., Robert S., Duy P.Q., Mermin-Bunnell N., Reeves B.C., Kundishora A., Elsamadicy A.A., Smith H. (2021). PTEN Mutations in Autism Spectrum Disorder and Congenital Hydrocephalus: Developmental Pleiotropy and Therapeutic Targets. Trends Neurosci..

[164] Srivastava S., Jo B., Zhang B., Frazier T., Gallagher A.S., Peck F., Levin A.R., Mondal S., Li Z., Filip-Dhima R. (2022). A Randomized Controlled Trial of Everolimus for Neurocognitive Symptoms in PTEN Hamartoma Tumor Syndrome. Hum. Mol. Genet..

[165] Overwater I.E., Rietman A.B., Mous S.E., Bindels-de Heus K., Rizopoulos D., Ten Hoopen L.W., van der Vaart T., Jansen F.E., Elgersma Y., Moll H.A. (2019). A Randomized Controlled Trial with Everolimus for IQ and Autism in Tuberous Sclerosis Complex. Neurology.

